# Jatrophane Diterpenoids from *Euphorbia peplus* with LysoTracker-Associated Cellular Activity

**DOI:** 10.3390/molecules31173024

**Published:** 2026-08-28

**Authors:** Mingrui Yuan, Shipeng Guan, Bowen Ouyang, Zifei Xu, Peng Wang, Qing Zhao, Chonglin Yang, Xiaojiang Hao, Qiuyuan Yin, Yingtong Di

**Affiliations:** 1 State Key Laboratory of Phytochemistry and Natural Medicines, Kunming Institute of Botany, Chinese Academy of Sciences, Kunming 650201, China; 2 University of Chinese Academy of Sciences, Beijing 100049, China; 3 School of Chinese Materia Medica, Yunnan University of Traditional Chinese Medicine, Kunming 650500, China; 4 Science and Technology Information Center, Kunming Institute of Botany, Chinese Academy of Sciences, Kunming 650201, China; 5 Yunnan Key Laboratory of Natural Medicinal Chemistry, Yunnan University, Kunming 650091, China

**Keywords:** *Euphorbia peplus*, jatrophane diterpenoids, LysoTracker Green, lysosome-associated fluorescence, phenotypic screening

## Abstract

Dysregulation of the autophagy–lysosome pathway is implicated in neurodegenerative disorders. To extend the chemical diversity of jatrophane diterpenoids from *Euphorbia peplus* and evaluate lysosome-associated phenotypes, phytochemical isolation and a LysoTracker Green assay in HeLa cells were performed. Five previously undescribed jatrophane diterpenoids, Euphjatrophanes M–Q (**1**–**5**), together with three known analogues (**6**–**8**), were isolated. The structures of **1**–**5** were assigned from one- and two-dimensional NMR, IR, and UV and the available HR-ESI-MS data, together with a comparison with reported compounds. At 10 and 40 μM, compounds **1**, **2**, and **4**–**8** produced higher LysoTracker-associated fluorescence than the DMSO vehicle control, whereas compound **3** showed a small numerical increase at 10 μM and a decrease at 40 μM. Compound **1** gave the largest numerical response (1.76-fold and 3.01-fold at 10 and 40 μM, respectively). These findings provide preliminary cell-based phenotypic evidence of lysosome-modulating activity and require orthogonal validation.

## 1. Introduction

The autophagy–lysosome pathway is an evolutionarily conserved intracellular degradation and recycling system that contributes to cellular homeostasis by removing damaged organelles, misfolded proteins, and other cytoplasmic material [1,2]. Dysfunction of this pathway has been associated with several neurodegenerative disorders, including Alzheimer’s disease (AD). Impaired lysosomal clearance may contribute to the accumulation of amyloid-β and hyperphosphorylated tau and thereby promote neuronal dysfunction [3]. These observations have motivated the investigation of small molecules that modulate cellular phenotypes related to autophagy and lysosomes [4].

Diterpenoids are structurally diverse specialized metabolites of the genus *Euphorbia* and have been reported to affect cellular processes associated with autophagy and lysosomes [5,6,7]. *Euphorbia peplus* contains numerous oxygenated diterpenoids, including jatrophane, lathyrane, paraliane, ingenane, pepluane, and tigliane congeners. Their scaffolds bear variable hydroxy, acetyl, benzoyl, nicotinoyl, and other acyl substituents, resulting in considerable chemical diversity [8,9,10,11,12,13,14,15,16,17,18,19]. Euphorbia diterpenoids have also been investigated for anti-tumor, anti-inflammatory, antimicrobial, multidrug-resistance-modulating, and other biological effects [17,18,20,21,22,23,24,25,26,27,28].

Our previous phytochemical studies of *E. peplus* yielded Euphjatrophanes A–L and diterpenoids with several other carbon skeletons [29,30,31,32,33,34,35,36]. In the present study, chromatographic separation of the whole plant afforded five previously undescribed jatrophane diterpenoids, Euphjatrophanes M–Q (**1**–**5**), and three known analogues (**6**–**8**). The new compounds were characterized using optical rotation, UV, IR, available HR-ESI-MS data, one-dimensional NMR, and two-dimensional NMR experiments, including HSQC, ^1^H–^1^H COSY, HMBC, and ROESY. Compounds **1**–**8** were then examined at two concentrations in a LysoTracker Green fluorescence assay in HeLa cells. The cells were used as a phenotypic screening platform for preliminary bioactivity evaluation in this study, rather than serving as a neuronal cell or neurodegenerative disease model. Therefore, the biological observations obtained here should not be directly extrapolated to pathological conditions of the nervous system. Because this assay provides a single fluorescence-based cellular endpoint, the resulting changes are interpreted as preliminary lysosome-associated phenotypes rather than evidence of lysosomal biogenesis, TFEB activation, autophagic-flux enhancement, or therapeutic activity.

## 2. Results

**Figure 1:**
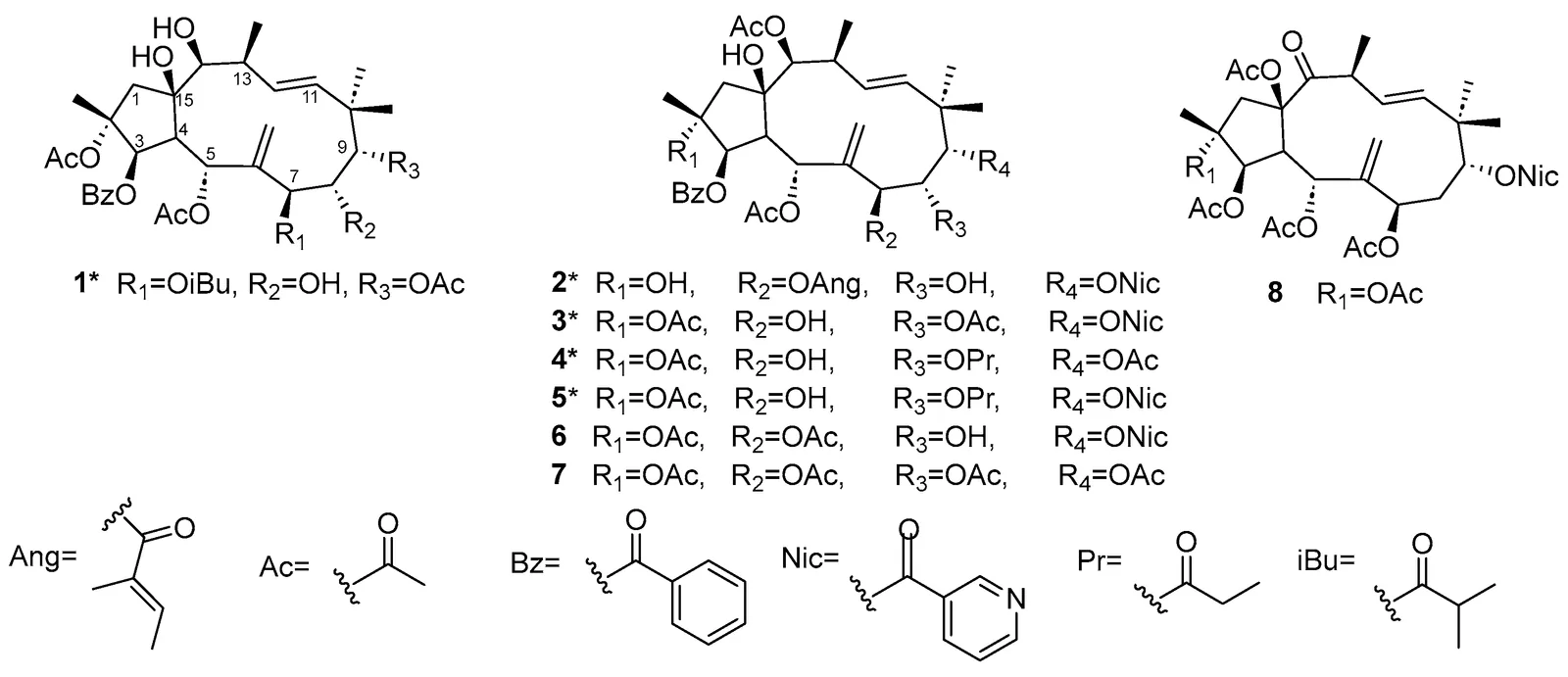
Structures of compounds **1**–**8**. Compounds marked with an asterisk (*) are previously undescribed. Bz, benzoyl; iBu, isobutyryl; Nic, nicotinoyl; Ang, angeloyl; Ac, acetyl; Pr, propionyl.

Five previously undescribed jatrophane diterpenoids, Euphjatrophanes M–Q (**1**–**5**), together with three known analogues (**6**–**8**), were isolated from *Euphorbia peplus* (Figure 1). Their planar structures and proposed relative configurations were assigned using the available HR-ESI-MS, one- and two-dimensional NMR, IR, and UV data.

### 2.1. Structure Elucidation

**Figure 2:**
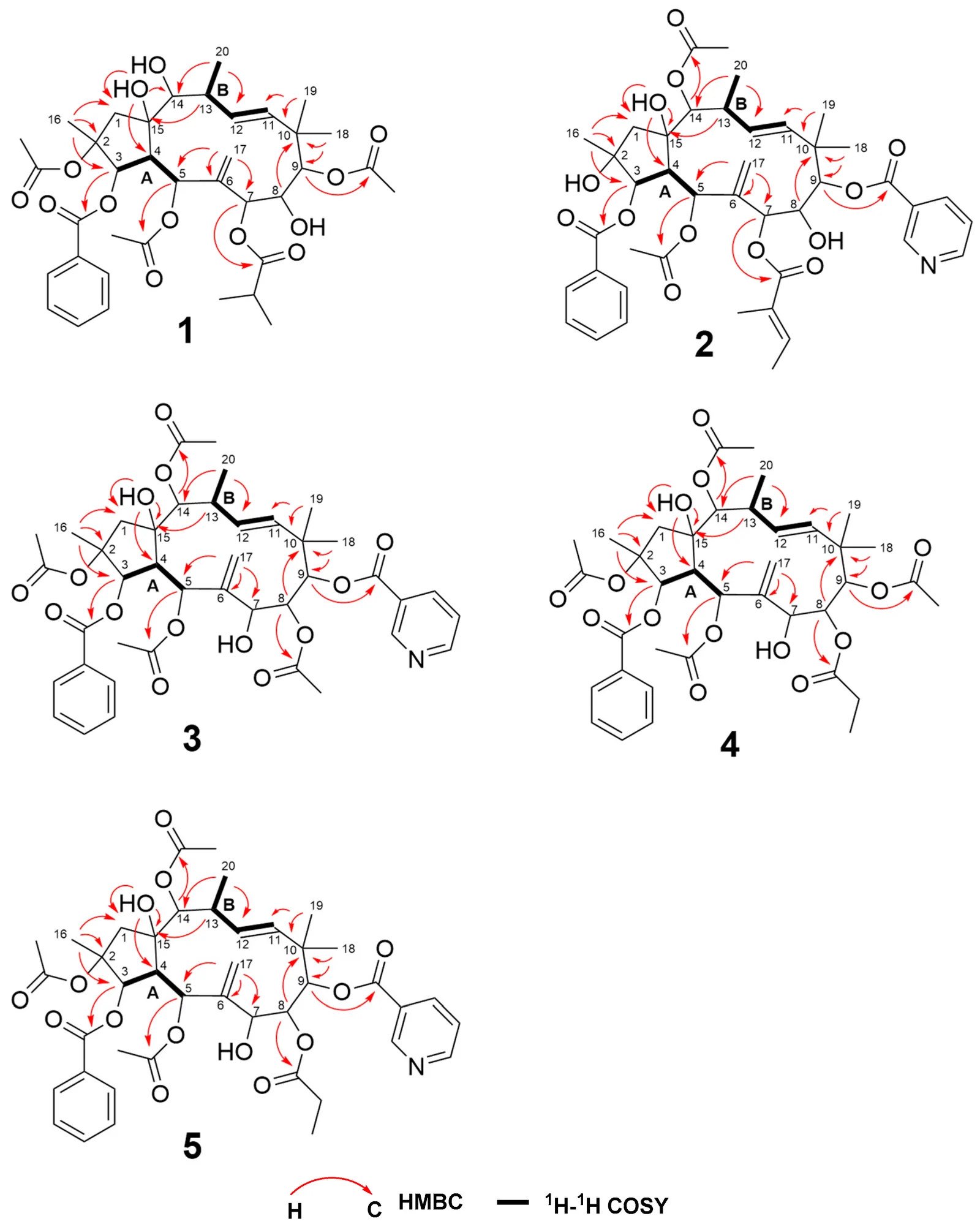
Key ^1^H–^1^H COSY and HMBC correlations of Euphjatrophanes M–Q (**1**–**5**).

**Figure 3:**
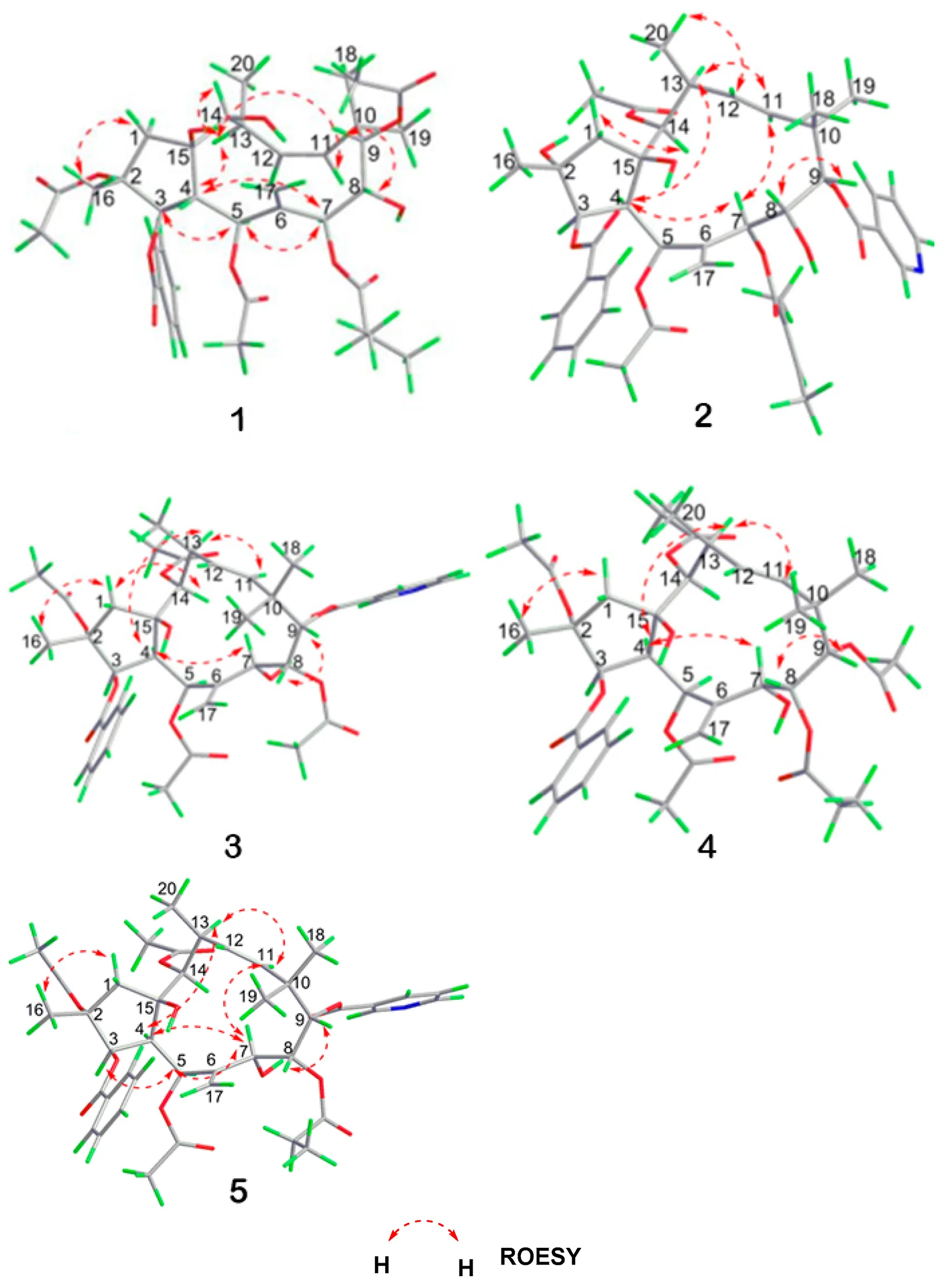
Key ROESY correlations of Euphjatrophanes M–Q (**1**–**5**). green for hydrogen atoms, red for oxygen atoms, gray for carbon atoms, and blue for nitrogen atoms.

Euphjatrophane M (**1**) was obtained as a pale yellow amorphous powder (MeOH). Its neutral molecular formula was determined as C_37_H_50_O_13_. The positive-ion HR-ESI-MS displayed a sodiated molecular ion peak at *m*/*z* 725.3136 [M + Na]^+^ (calcd 725.3144 for C_37_H_50_O_13_Na), corresponding to 13 degrees of unsaturation. IR absorptions indicated hydroxy, carbonyl, and benzoyl functionalities. The ^1^H NMR (Figure S1), ^13^C NMR (Figure S2), and HSQC (Figure S4) data (Table 1) showed three acetyl, one benzoyl, and one isobutyryl group, together with two exchangeable protons. Five ester carbonyl resonances were observed. After excluding the acyl-substituent carbons, the remaining 20 signals were consistent with a jatrophane carbon skeleton (Figure 1). The acyl groups and two double bonds accounted for 11 degrees of unsaturation, leaving two for the bicyclic core. HMBC correlations (Figure S5) from H-3 to the carbonyl carbon at *δ*_C_ 164.7, H-5 to *δ*_C_ 168.5, H-7 to *δ*_C_ 174.8, and H-9 to *δ*_C_ 172.0 supported the placement of benzoyl at C-3, acetyl at C-5, isobutyryl at C-7, and acetyl at C-9, respectively (Figure 2). The spectroscopic data resembled those of Euphjatrophane J [36], with the main reported difference being the replacement of its C-9 nicotinoyl group by an acetyl group. Through-space ROESY correlations (Figure S6)assigned in the manuscript as H-4α/H-7/H-13, H-3/H-7, and H-11/H-13 were used to propose a common molecular face for these protons (Figure 3). The relative configurations of H-2, H-5, H-8, H-9, H-14, and H-15 were assigned by analogy with literature data [36]. Accordingly, the relative configuration of **1** was proposed as (2R*, 3R*, 4S*, 5S*, 7S*, 8R*, 9S*, 13S*, 14S*, 15R*)-2,5,9-triacetoxy-8,14,15-trihydroxy-7-isobutyryloxy-3-benzoyloxyjatropha-6(17),11E-diene (Figure 1).

Euphjatrophane N (**2**) was isolated as a white amorphous powder (MeOH). Its neutral molecular formula was determined as C_42_H_51_NO_13_. The positive-ion HR-ESI-MS exhibited a protonated molecular ion peak at *m*/*z* 778.3443 [M + H]^+^ (calcd 778.3433 for C_42_H_52_NO_13_), corresponding to 18 degrees of unsaturation. The IR spectrum showed bands attributable to hydroxy, ester-carbonyl, and alkene functionalities. The ^1^H (Figure S11) and ^13^C NMR (Figure S12) data (Table 1) indicated two acetyl, one benzoyl, one angeloyl, and one nicotinoyl substituent, together with two exchangeable protons. Four olefinic protons were assigned to one trans-disubstituted and one 1,1-disubstituted double bond. The data were similar to those reported for euphpepluone K [13], with the proposed differences being a C-2 hydroxy group in place of acetoxy and a C-7 angeloyl group in place of tigloyl (Figure 2). The reported ROESY correlations (Figure S16) H-4α/H-7/H-13 and H-11/H-13 supported a common face for H-7, H-11, and H-13 (Figure 3). The relative configurations of H-2, H-3, H-5, H-8, H-9, H-14, and H-15 were assigned by analogy with literature data [13]. The relative structure of **2** was proposed as (2R*, 3R*, 4S*, 5S*, 7S*, 8R*, 9S*, 13S*, 14S*,15R*)-5,14-diacetoxy-2,8,15-trihydroxy-7-angeloyloxy-3-benzoyloxy-9-nicotinoyloxyjatropha-6(17),11E-diene (Figure 1).

Euphjatrophane O (**3**) was obtained as a white amorphous powder (MeOH). Its neutral molecular formula was determined as C_41_H_49_NO_14_. The positive-ion HR-ESI-MS exhibited a protonated molecular ion peak at *m*/*z* 780.3232 [M + H]^+^ (calcd 780.3226 for C_41_H_50_NO_14_), corresponding to 18 degrees of unsaturation. IR bands indicated hydroxy, carbonyl, and aromatic functionalities. The ^1^H (Figure S21) and ^13^C NMR (Figure S22) data (Table 2) showed four acetyl, one benzoyl, and one nicotinoyl moiety, corresponding to six ester-carbonyl carbons. HMBC correlations (Figure S25) supported the C-1/C-2/C-3/C-4/C-15 five-membered ring and double bonds at Δ^6^(17) and Δ^11^(12). The acyl substituents and two olefinic bonds accounted for 16 degrees of unsaturation, leaving two for the bicyclic jatrophane scaffold. Relative to pepluanin A [37], **3** was proposed to contain a C-7 hydroxy group in place of acetoxy. The HMBC correlations from H-17 (δH 4.83) to C-7 (δC 67.2) were consistent with this assignment (Figure 2). The reported ROESY correlations (Figure S26) H-4α/H-7/H-13 and H-11/H-13 supported a common face for these protons (Figure 3). The relative configurations of H-2, H-3, H-5, H-8, H-9, H-14, and H-15 were assigned by analogy with literature data [37]. The relative structure was proposed as (2R*, 3R*, 4S*, 5S*, 7S*, 8R*, 9S*, 13S*, 14S*, 15R*)-2,5,8,14-tetraacetoxy-7,15-dihydroxy-3-benzoyloxy-9-nicotinoyloxyjatropha-6(17),11E-diene (Figure 1).

Euphjatrophane P (**4**) was isolated as a pale-yellow amorphous powder (MeOH). Its neutral molecular formula was determined as C_38_H_50_O_14_. The positive-ion HR-ESI-MS displayed a sodiated molecular ion peak at *m*/*z* 753.3093 [M + Na]^+^ (calcd 753.3093 for C_38_H_50_O_14_Na), corresponding to 14 degrees of unsaturation. The ^1^H (Figure S31) and ^13^C NMR (Figure S32) data (Table 2) showed four acetyl groups, one benzoyl group, and one propionyl group. After exclusion of the acyl-substituent signals, the 20 carbon resonances of the diterpene core comprised four methyl, two methylene, ten methine, and four quaternary carbons. These functionalities accounted for 12 degrees of unsaturation, leaving two for the bicyclic scaffold. The data resembled those of Euphjatrophane D [6], with a propionyl group proposed at C-8 in place of the isobutyryl group (Figure 2). The reported ROESY correlations (Figure S36) H-4α/H-7/H-13 and H-11/H-13 supported a common face for these protons (Figure 3). The relative configurations of H-2, H-3, H-5, H-8, H-9, H-14, and H-15 were assigned by analogy with literature data [6]. The relative structure was proposed as (2R*, 3R*, 4S*, 5S*, 7S*, 8R*, 9S*, 13S*, 14S*, 15R*)-2,5,9,14-tetraacetoxy-7,15-dihydroxy-3-benzoyloxy-8-propionyloxyjatropha-6(17),11E-diene (Figure 1).

Euphjatrophane Q (**5**) was obtained as a white gelatinous solid. Its neutral molecular formula was determined as C_42_H_51_NO_14_. The positive-ion HR-ESI-MS exhibited a protonated molecular ion peak at *m*/*z* 794.3380 [M + H]^+^ (calcd 794.3382 for C_42_H_52_NO_14_), corresponding to 18 degrees of unsaturation. The ^1^H (Figure S41) and ^13^C NMR (Figure S42) data (Table 2) indicated three acetyl, one benzoyl, one propionyl, and one nicotinoyl group. After exclusion of the acyl-substituent signals, the 20 carbon resonances of the diterpene core comprised four methyl, two methylene, ten methine, and four quaternary carbons. Compound **5** was closely related to **4**, but the C-9 acetyl group of **4** was replaced by a nicotinoyl group in **5** (Figure 1 and Figure 2). The reported ROESY correlations (Figure S46) of H-4 with H-7/H-13, H-3/H-5 with H-7, and H-11 with H-13 were used to assign the relative configuration (Figure 3). The relative configurations of H-2, H-8, H-9, H-14, and H-15 were assigned by analogy with literature data [6]. The relative structure was proposed as (2R*, 3R*, 4S*, 5S*, 7S*, 8R*, 9S*, 13S*, 14S*, 15R*)-2,5,14-triacetoxy-7,15-dihydroxy-3-benzoyloxy-8-propionyloxy-9-nicotinoyloxyjatropha-6(17),11E-diene (Figure 1).

The known compounds were identified as (2R*, 3R*, 4S*, 5R*, 7S*, 8S*, 9S*, 13S*, 14S*, 15R*)-2,5,9,14-tetraacetoxy-3-benzoyloxy-8,15-dihydroxy-7-isobutyryloxyjatropha-6(17),11E-diene (**6**) [38], (2R*, 3R*, 4S*, 5R*, 7S*, 8S*, 9S*, 13S*, 14S*, 15R*)-2,5,7,8,9,14-hexaacetoxy-3-benzoyloxy-15-hydroxyjatropha-6(17),11E-diene (**7**) [38], and (2R*, 3R*, 4S*, 5R*, 7R*, 9R*, 13R*, 15R*)-2,3,5,7,15-pentaacetoxy-9-nicotinoyloxy-14-oxojatropha-6(17),11E-diene (**8**) [39]. Their identities were assigned by comparison of MS and NMR data with the cited literature (Figure 1).

### 2.2. Bioactivity Evaluation and Preliminary Structure Response Observations

**Figure 4:**
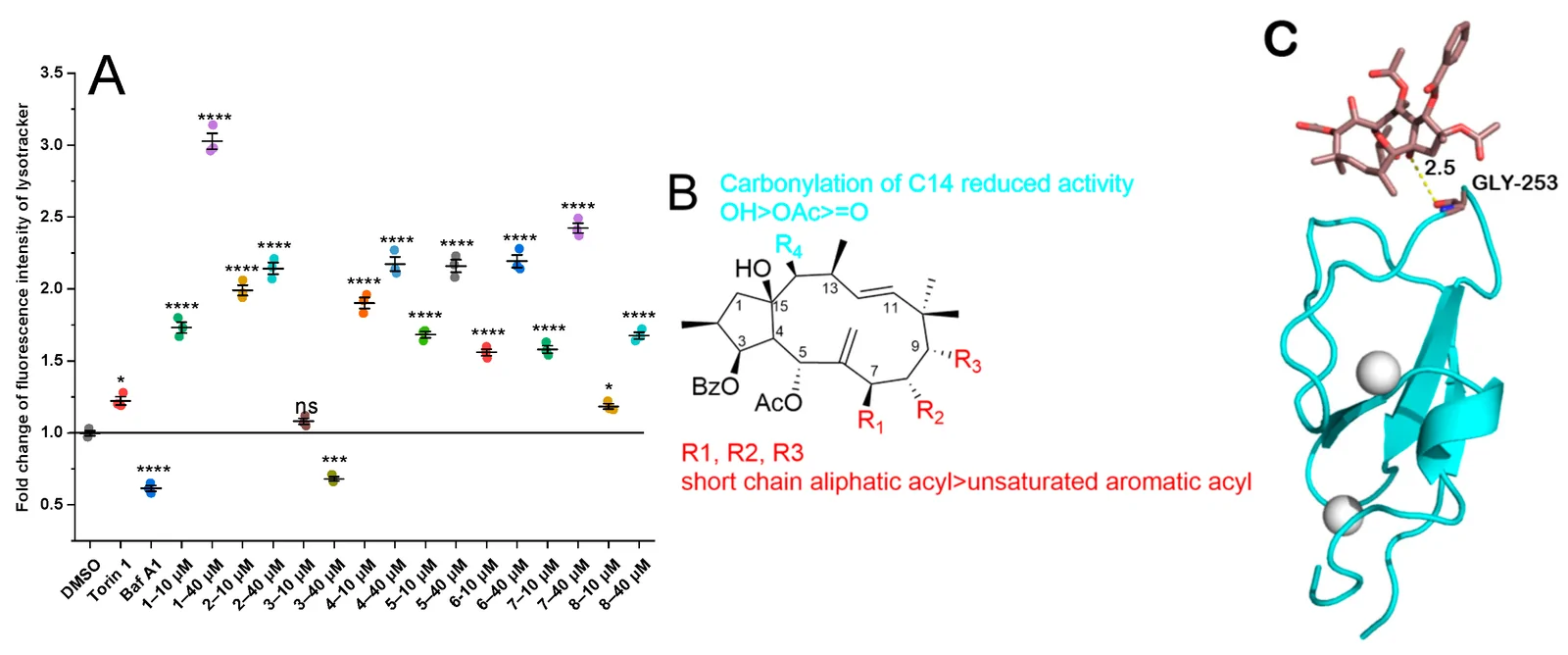
(**A**) Fold change in LysoTracker Green fluorescence after treatment with compounds **1**–**8**. HeLa cells treated with DMSO served as the vehicle control; Torin 1 (1 μM) and bafilomycin A1 (Baf A1; 1 μM) were included as assay controls. Values are reported as the mean ± standard deviation from three independent experiments (*n* = 3). Statistical significance relative to the DMSO control was evaluated using one-way ANOVA followed by Dunnett’s multiple-comparison test: * *p* < 0.05, *** *p* < 0.001, **** *p* < 0.0001; ns, not significant. (**B**) Exploratory qualitative comparison of structural features and LysoTracker Green fluorescence responses. Data shown in panel B represent working hypotheses derived from eight nonmatched compounds in which multiple substituents varied simultaneously. (**C**) Molecular docking analysis of Euphjatrophane M (compound **1**) against the C1 domain of PKC (PDB: 7KO6) [48]. Oxygen and nitrogen atoms are color-coded in red and blue, respectively. Compound **1** is shown in brown-red. The PKC structure is presented in cartoon representation, and the key residue GLY-253 is shown as brown sticks. Hydrogen bonds between compound **1** and the C1 domain are depicted as yellow dashed lines.

The LysoTracker Green fluorescence assay in HeLa cells was used to compare lysosome-associated phenotypes for compounds **1**–**8**. DMSO was used as the vehicle control, whereas Torin 1 and bafilomycin A1 (Baf A1) were set as positive and negative assay controls, respectively. Fluorescence intensity was normalized to the DMSO-treated group and presented as fold change (Figure 4A). Notably, cell viability and cell number were not determined in parallel under identical assay conditions, which represents an important methodological limitation, particularly given the maximum test concentration of 40 μM. For instance, compound **3** exhibited a fold-change value of 1.09 at 10 μM and 0.69 at 40 μM. We cannot rule out the possibility that the reduced LysoTracker Green signal at 40 μM arises from cytotoxic effects or decreased cell counts rather than genuine lysosome-related alterations. In addition, compound-derived intrinsic fluorescence or fluorescence quenching was not assessed in the present dataset. Therefore, alterations in LysoTracker Green fluorescence should not be directly interpreted as quantitative readouts of lysosome abundance, lysosomal function, or lysosomal biogenesis.

Jatrophane diterpenoids have been reported to display diverse biological activities, including multidrug-resistance modulation, anti-tumor, anti-viral, anti-inflammatory, anti-arrhythmic, anti-bacterial, neuroprotective, and lysosome-associated effects [40,41,42,43,44,45,46]. The present experiment, however, was designed only as a LysoTracker-based phenotypic screen and did not test these mechanisms or therapeutic effects.

Relative to the DMSO control, compound **1** produced 1.76-fold and 3.01-fold fluorescence at 10 and 40 μM, respectively, and gave the largest numerical response among the eight compounds. Compound **2** produced a significant increase at 10 μM (approximately 1.98-fold) and 2.16-fold fluorescence at 40 μM. Compound **3** produced 1.09-fold fluorescence at 10 μM (not significant) but a lower signal at 40 μM (0.69-fold; *p* < 0.001); this decrease was recorded as a fluorescence phenotype and was not interpreted as inhibition of lysosomal activity. Compounds **4** and **5** produced 1.90- and 2.18-fold and 1.68- and 2.15-fold fluorescence at 10 and 40 μM, respectively. The corresponding values for compounds **6** and **7** were 1.54 and 2.19 and 1.59 and 2.42. Compound **8** produced a modest increase at 10 μM (1.19-fold) and a significant, larger increase at 40 μM (Figure 4A).

Panel B summarizes preliminary qualitative observations intended to guide future work. The eight compounds do not constitute rigorously matched pairs, and the dataset is too small to establish a causal substituent–response relationship or a definitive rank order such as OH > OAc ≥ O. Likewise, the data do not establish that short-chain acyl groups are intrinsically more favorable than nicotinoyl groups. These comparisons should therefore be treated as working hypotheses requiring larger analogue series, dose–response analysis, cell-viability and cell-number controls, fluorescence-interference controls, and orthogonal lysosomal assays.

Previous studies have reported that ingenane-type diterpenoids trigger autophagy via binding to the C1 domain of PKCδ [47]. Herein, molecular docking was performed to further characterize the interaction of compound **1**, which exhibited the most prominent LysoTracker-associated cellular activity, with the PKCδ C1 domain. Docking simulations (Figure 4C) revealed that the oxygen atom of the C-15 hydroxyl group in compound **1** forms a hydrogen bond with GLY253. The hydrophobic fragments consisting of one five-membered ring and one twelve-membered ring within the skeleton of compound **1** are positioned at the surface of the binding domain, which facilitates insertion of the ligand into the phospholipid bilayer. For compounds **2**–**7**, acetylation of the C-14 hydroxyl group alters the molecular planar conformation and prevents proper anchoring of the molecule onto the surface of the protein-binding domain; consequently, the C-15 hydroxyl oxygen fails to establish a productive hydrogen-bond interaction with GLY253. In contrast, compound **1**, lacking C-14 acetylation, maintains the optimal spatial proximity between PKC and the cell membrane. As for compound **8**, direct acetylation at the C-15 position abolishes the hydroxyl group required for binding to the key residue GLY253. It should be emphasized that all mechanistic inferences derived from the above molecular docking analysis represent computational hypotheses only, which require further experimental validation and cannot be regarded as definitive biological conclusions.

## 3. Discussion

Five previously undescribed jatrophane diterpenoids and three known analogues were isolated from *Euphorbia peplus*. The planar structures and relative configurations of compounds **1**–**5** were assigned based on comprehensive spectroscopic data and structural comparison with previously reported congeners, representing the distinct phytochemical novelty of the present study. In the LysoTracker Green phenotypic assay, several isolates markedly increased lysosome-associated fluorescence relative to the vehicle control, with compound **1** exhibiting the most prominent elevation, whereas compound **3** displayed reduced fluorescence intensity at 40 μM. Importantly, alterations in LysoTracker Green fluorescence are not exclusively indicative of lysosomal biological regulation, as multiple confounding factors may interfere with the assay readout. These alternative variables include changes in lysosomal acidification status, fluctuations in acidic-compartment volume, variations in cell number, altered cellular uptake or intracellular retention of the fluorescent probe, compound-derived fluorescence interference, and potential cytotoxicity induced by high compound concentrations. Collectively, the present biological findings should be strictly considered preliminary phenotypic screening results rather than definitive functional validation. Notably, although the phytochemical identification of five new jatrophane diterpenoids constitutes the principal novelty of this work, the biological contribution of the current study remains exploratory and preliminary, without definitive evidence to confirm lysosomal biogenesis, TFEB activation, autophagic flux enhancement, or neuroprotective activity of the tested compounds.

The interpretation is limited by the use of a single cell line, one fluorescence-based readout, and only two tested concentrations. Importantly, these experiments lacked parallel controls for cell viability, cell number, and compound-related fluorescence interference. As exemplified by compound **3**, which exhibited a 1.09-fold signal at 10 μM and a 0.69-fold signal at 40 μM, we cannot exclude the notion that reduced LysoTracker Green fluorescence at higher concentrations may result from cytotoxicity-triggered cell loss rather than bona fide lysosome-related phenotypes. Furthermore, the limited panel of nonmatched analogues precludes drawing definitive conclusions regarding structure–activity relationships. Future investigations should incorporate full dose–response profiling, parallel cytotoxicity and cell-quantification measurements, normalization of fluorescence signals to viable cell counts where possible, orthogonal readouts for lysosomal abundance and function, and autophagic-flux assays, as well as mechanistic assays such as TFEB nuclear localization and downstream target gene expression analysis.

To obtain preliminary mechanistic clues for the observed phenotypic responses, we carried out molecular docking simulations against the PKCδ C1 domain. Nevertheless, these docking-predicted interaction modes represent purely in silico hypotheses. Direct experimental proof of physical compound-target binding remains absent, and further biochemical investigations are thus needed to validate these computationally predicted interactions.

## 4. Materials and Methods

### 4.1. General Experimental Procedures

Optical rotations were measured on a Jasco P-1020 automatic polarimeter (Jasco Corporation, Tokyo, Japan) using a 5 cm cell. UV spectra were recorded on an Applied Photophysics circular dichroism spectrometer (Applied Photophysics Ltd., Leatherhead, UK) using a 0.1 cm path-length cell. IR spectra were recorded on a Nicolet iS10 Fourier-transform infrared spectrometer (Thermo Scientific, Waltham, MA, USA). HR-ESI-MS spectra were obtained on an Agilent 6545 Q-TOF mass spectrometer (Agilent Technologies Inc., Santa Clara, CA, USA). NMR experiments were performed on a Bruker Avance DRX500 spectrometer (Bruker BioSpin AG, Fällanden, Switzerland), operating at 500 MHz for ^1^H and 125 MHz for ^13^C, with TMS as the internal standard. Reversed-phase HPLC separations were performed on an Agilent 1260 series LC instrument (Agilent Technologies Inc., Santa Clara, CA, USA) equipped with a Waters XBridge BEH C18 T3 column (250 × 10 mm, 5 μm; Waters Corporation, Milford, MA, USA). Column chromatography (CC) was performed using silica gel (100–200, 200–300, or 300–400 mesh; Qingdao Haiyang Chemical Co., Ltd., Qingdao, China). LiChroprep RP-18 (40–63 μm; Merck KGaA, Darmstadt, Germany) was used for reversed-phase CC. Precoated silica gel GF254 plates (Qingdao Haiyang Chemical Co., Ltd., Qingdao, China) were used for TLC. Solvents used for CC were of analytical grade (Shanghai Macklin Biochemical Co., Ltd., Shanghai, China). Sephadex LH-20 (20–150 μm; Pharmacia Corporation, Uppsala, Sweden) was used for CC. HPLC-grade solvents were obtained from Merck KGaA (Darmstadt, Germany).

### 4.2. Plant Material

The whole plant of *Euphorbia peplus* was collected in July 2020 from Kunming Botanical Garden, Kunming Institute of Botany, Yunnan Province, China. The material was identified by Prof. Shi-Jun Hu (Southwest Forestry University). A voucher specimen (No. Kep-09-18) was deposited in the herbarium of the Kunming Institute of Botany, Chinese Academy of Sciences.

### 4.3. Extraction and Isolation

Air-dried and powdered whole plants of *E. peplus* (20.0 kg) were extracted with methanol (3 × 30 L) at room temperature for 36 h per extraction. The combined extracts were concentrated under reduced pressure to give a crude residue (3.0 kg). The residue was partitioned three times between ethyl acetate and water (1:1, *v*/*v*; 20 L per partition), and the ethyl acetate layer was concentrated (0.6 kg). The residue was adsorbed onto silica gel (1.2 kg), applied to a silica-gel column, and eluted with a petroleum ether/ethyl acetate gradient (100:0 to 0:100, *v*/*v*) to give fractions F1–F4. Fraction F2 (201 g) was separated by MCI gel CHP 20P CC using a stepwise MeOH/H_2_O gradient (40:60 to 100:0, *v*/*v*) to give fractions F2-1-F2-6. F2-1 (31.3 g) was separated by silica-gel CC using petroleum ether/ethyl acetate (20:1 to 1:1, *v*/*v*) to give subfractions F2-1-3 (20.4 mg), which were purified by preparative HPLC with CH_3_CN/H_2_O (50:50, *v*/*v*) at 2.0 mL/min to yield **1** (12.1 mg) and **2** (3.2 mg). Fraction F2-5 (22.3 g) was separated by silica-gel CC with a CH_2_Cl_2_/MeOH gradient (100:0 to 0:100, *v*/*v*) to give subfractions F2-5-1–F2-5-5. F2-5-4 (34.3 mg) was separated by Sephadex LH-20 CC with CH_2_Cl_2_/MeOH (50:50, *v*/*v*) to give subfractions F2-5-4-1–F2-5-4-5. F2-5-4-5 (22.7 mg) was purified by semipreparative HPLC with CH_3_CN/H_2_O (60:40, *v*/*v*) to yield **3** (7.5 mg) and **4** (5.3 mg), and F2-5-4-4 (17.7 mg) was purified by semipreparative HPLC with CH_3_CN/H_2_O (70:30, *v*/*v*) to yield **5** (6.7 mg). Subfraction F2-5-5 (46.9 mg) was purified by normal-phase preparative HPLC on a CHIRALPAK^®^ AD-H column (10 × 250 mm, 5 μm) using n-hexane/isopropanol (40:60, *v*/*v*) to afford compounds **6** (7.6 mg) and **7** (8.2 mg). The residual eluate of this chiral separation was pooled and concentrated to obtain subfraction F2-5-5-2 (19.7 mg), which was further purified by semipreparative HPLC with CH_3_CN/H_2_O (40:60, *v*/*v*) to yield compound **8** (6.8 mg).

### 4.4. Compound Characterization

Euphjatrophane M (**1**)

Pale-yellow amorphous powder; ${\left[\alpha \right]}_{D}^{24}$ = −6.1 (c 0.16, MeOH); UV (MeOH) λ_max_ (log ε) 229 (3.07) nm; IR (KBr) *ν*_max_ 3451, 2971, 1733, 1376, 1245, 1145, 1113, 1027 cm^−1^; CD (MeOH) λ (∆ε) = 195 (−17.06), 204 (2.05), 222 (−1.03); for ^1^H and ^13^C NMR data, see Table 1; HR-ESI-MS: *m*/*z* 725.3136 [M + Na]^+^ (calcd for C_37_H_50_NaO_13_, 725.3144). The measured mass value was consistent with the theoretical calculation, with a mass error less than 1.1 ppm.

Euphjatrophane N (**2**)

White amorphous powder; ${\left[\alpha \right]}_{D}^{24}$ = +90.0 (c 0.23, MeOH); UV (MeOH) λ_max_ (log ε) 225 (3.28) nm; IR (KBr) *ν*_max_ 3444, 2970, 2932, 1726, 1593, 1377, 1277, 1146, 1114, 1024 cm^−1^; CD (MeOH) λ (∆ε) = 195 (8.12), 201 (10.01), 219 (−3.01), 235 (6.66); for ^1^H and ^13^C NMR data, see Table 1; HR-ESI-MS: *m*/*z* 778.3443 [M + H]^+^ (calcd for C_42_H_52_NO_13_, 778.3433). The measured mass value was consistent with the theoretical calculation, with a mass error less than 1.28 ppm.

Euphjatrophane O (**3**)

White amorphous powder; ${\left[\alpha \right]}_{D}^{24}$ = +33.0 (c 0.24, MeOH); UV (MeOH) λ_max_ (log ε) 225 (3.15) nm; IR (KBr) *ν*_max_ 3579, 3443, 2969, 2933, 1726, 1593, 1376, 1280, 1236, 1026 cm^−1^; CD (MeOH) λ (∆ε) = 195 (−0.076), 203 (4.67), 218 (5.04); for ^1^H and ^13^C NMR data, see Table 2. HR-ESI-MS: *m*/*z* 780.3232 [M + H]^+^ (calcd for C_41_H_50_NO_14_, 780.3226). The measured mass value was consistent with the theoretical calculation, with a mass error less than 0.77 ppm.

Euphjatrophane P (**4**)

Pale-yellow amorphous powder; ${\left[\alpha \right]}_{D}^{24}$ = −11.6 (c 0.21, MeOH); UV (MeOH) λ_max_ (log ε) 230 (2.85) nm; IR (KBr) *ν*_max_ 3448, 2966, 2927, 1746, 1375, 1233, 1112, 1071 cm^−1^; CD (MeOH) λ (∆ε) = 195 (−20.79), 205 (2.22), 225 (−0.98); for ^1^H and ^13^C NMR data, see Table 2; HR-ESI-MS: *m*/*z* 753.3093 [M + Na]^+^ (calcd for C_38_H_50_NaO_14_, 753.3093). The experimental mass was consistent with the theoretically calculated value.

Euphjatrophane Q (**5**)

White gelatinous solid; ${\left[\alpha \right]}_{D}^{24}$ = +20.8 (c 0.17, MeOH); UV (MeOH) λ_max_ (log ε) 226 (2.88) nm; IR (KBr) *ν*_max_ 3579, 3442, 2924, 2853, 1728, 1377, 1277, 1235, 1115, 1025 cm^−1^; CD (MeOH) λ (∆ε) = 195 (−1.38), 203 (4.58), 218 (3.43); for ^1^H and ^13^C NMR data, see Table 2; HR-ESI-MS: *m*/*z* 794.3380 [M + H]^+^ (calcd for C_42_H_52_NO_14_, 794.3382). The measured mass value was consistent with the theoretical calculation, with a mass error less than 0.25 ppm.

### 4.5. Cell Culture and Bioactivity Assessment

Human cervical carcinoma HeLa cells (ATCC CCL-2) were used for phenotypic screening of LysoTracker-associated fluorescence. Cells were maintained in DMEM supplemented with 10% fetal bovine serum and 1% penicillin–streptomycin at 37 °C in a humidified atmosphere containing 5% CO_2_. Cells in logarithmic growth were seeded into 96-well plates at a density of 1.2 × 10^4^ cells per well with a final well volume of 100 μL. At 80–90% confluence, cells were treated for 3 h with compounds **1**–**8** (10 or 40 μM), Torin 1 (1.0 μM), Baf A1 (1.0 μM), or DMSO vehicle. Torin 1 served as a positive control for autophagy induction, whereas Baf A1 was included as a reference compound to disrupt lysosomal acidification, an effect known to directly modulate LysoTracker Green dye accumulation within acidic compartments; the final DMSO concentration in all wells was kept at 0.1% (*v*/*v*). LysoTracker Green was then added to a final concentration of 50 nM, and the cells were incubated in the dark for 30 min. Cells were not washed prior to fluorescence measurement, and fluorescence acquisition was performed directly in the original culture medium. Fluorescence was recorded using a multifunctional microplate reader (Tecan Spark 20M, Tecan, Männedorf, Switzerland) with an excitation wavelength of 485 nm and an emission wavelength of 520 nm. Background subtraction was performed using blank wells containing medium without cells. Each experiment included three technical replicates per condition. The fluorescence signal for each treatment was normalized to the DMSO vehicle control value and reported as fold change.

### 4.6. Statistical Analysis

Quantitative data were analyzed using one-way analysis of variance followed by Dunnett’s multiple-comparison test in GraphPad Prism 8 (GraphPad Software, La Jolla, CA, USA), with comparisons made against the DMSO vehicle control group. Each independent biological experiment contained three technical replicates per condition. For each individual experiment, technical replicate values were averaged prior to statistical analysis. Data are reported as the mean ± standard deviation from three independent biological experiments (*n* = 3). Statistical significance is denoted as * *p* < 0.05, ** *p* < 0.01, *** *p* < 0.001, and **** *p* < 0.0001; ns, not significant.

### 4.7. Molecular Docking

The molecular docking study, including receptor preparation, ligand preparation, and docking calculation, was performed using Schrödinger’s Maestro 12.9 (Schrödinger, Inc., New York, NY, USA). The X-ray crystal structure of the PKCδ C1 domain was retrieved from the Protein Data Bank (PDB: 7KO6), available from the RCSB Protein Data Bank (RCSB PDB, Research Collaboratory for Structural Bioinformatics, Piscataway, NJ, USA; https://www.rcsb.org/). PyMOL version 3.1.1 (Schrödinger, Inc., New York, NY, USA) was applied to visualize docking outputs.

The downloaded PKCδ C1 domain structure was imported into Maestro and preprocessed via the Protein Preparation Wizard. Missing side chains were repaired, protonation states were corrected, and atomic clashes were eliminated. All crystallographic water molecules were removed, hydrogen-bond networks were optimized, and energy minimization was carried out to yield a stable receptor conformation.

Two-dimensional structures of target compounds were drawn in ChemDraw 22.0. Following preliminary conformational optimization within Chem3D 8.0, the structures were exported as three-dimensional mol2 files.

The Receptor Grid Generation module was utilized to construct a docking grid with a side length of 24 Å centered on the ligand. Molecular docking was executed using the Glide module with flexible ligand sampling enabled. Residue side chains located within 5 Å of the active-site pocket were set as flexible during docking.

A maximum of 20 binding poses were generated for each ligand. Obtained poses were pre-filtered using a GlideScore threshold of ≤−6.0 kcal/mol. The complex with the lowest binding energy was exported in PDB format for subsequent binding mode analysis.

## 5. Conclusions

Five previously undescribed jatrophane diterpenoids and three known analogues were isolated and identified from *Euphorbia peplus*. Several compounds altered LysoTracker-associated fluorescence in HeLa cells, with compound **1** giving the largest numerical increase; compound **3** showed a lower signal at 40 μM. These results constitute preliminary cell-based evidence that these compounds alter LysoTracker-associated fluorescence. Mechanistic studies, cytotoxicity and cell-number measurements, fluorescence-interference controls, and orthogonal validation of lysosomal abundance and function are required before the observed signals can be attributed to lysosomal biogenesis or translated into therapeutic claims.

## Figures and Tables

**Table 1:** ^1^H and ^13^C NMR data for compounds **1** and **2** in CDCl_3_ (*δ* in ppm, *J* in Hz).

No.	1 ^a^		2 ^a^	
	*δ*_H_	*δ*_C_	*δ*_H_	*δ*_C_
1α	2.53 m	51.3	2.25 d (14.9)	53.5
1β	2.11 s		2.13 d (14.7)	
2		87.9		78.4
3	5.83 m	80.9	5.58 d (3.8)	82.8
4	3.15 br d	44.3	3.41 m	44.1
5	5.75 br d	71.7	5.82 br s	71.4
6		144.8		143.9
7	5.28 s	68.1	5.46 s	69.7
8	4.05 m	70.0	4.15 br d	70.0
9	4.71 br s	86.3	5.04 s	87.1
10		40.0		40.3
11	5.82 br d	132.7	5.99 d (15.9)	133.2
12	5.96 dd (16.2, 9.4)	131.7	5.71 dd (15.9, 9.7)	132.1
13	2.51 m	37.7	2.90 m	36.7
14	3.63 br s	78.5	5.16 s	79.6
15		85.2		83.5
16	1.55 s	21.8	1.33 s	24.4
17	4.52 s; 4.92 s	109.3	4.92 s; 4.66 s	110.2
18	1.00 s	27.4	1.09 s	27.3
19	1.29 s	23.3	1.37 s	23.5
20	1.21 d (7.0)	24.6	1.20 d (6.5)	23.7
8-OH	3.03 d (10.9)		3.22 br d	
2-OAc	2.15 s	170.6		
		22.4		
3-OBz	8.07 m	164.7	8.03 d (7.4)	165.3
	7.46 m	130.0	7.43 t (7.4)	129.9
	7.59 m	129.6	7.57 m	129.6
		128.5		128.6
		133.3		133.3
5-OAc	1.97 s	168.5	2.11 s	168.8
		20.9		20.6
7-OiBu ^1^/7-OAng ^2^	2.55 m	174.8	5.80 m	165.7
	1.23 d (7.0)	33.9	1.72 d (8.3)	126.2
	1.19 d (7.0)	18.4	1.50 br s	142.4
		19.4		16.2
				20.0
9-OAc ^1^/9-ONic ^2^	2.04 s	172.0	9.18 br s	166.3
		20.8	8.24 m	151.2
			7.39 dd	124.9
			8.77 br d	137.2
				123.4
				153.8
14-OAc			1.87 s	170.8
				20.8

^a^ Recorded in CDCl_3_ at 500 MHz (^1^H) and 125 MHz (^13^C). s, singlet; d, doublet; t, triplet; dd, doublet of doublets; br s, broad singlet; br d, broad doublet; m, multiplet.Superscripts ^1^ and ^2^ represent Compound **1** and Compound **2**, respectively.

**Table 2:** ^1^H and ^13^C NMR data for compounds **3**–**5** in CDCl_3_ (δ in ppm, J in Hz).

No.	3 ^a^		4 ^a^		5 ^a^	
	*δ*_H_	*δ*_C_	*δ*_H_	*δ*_C_	*δ*_H_	*δ*_C_
1α	2.75 m	48.9	2.71 d (14.6)	49.1	2.76 m	48.8
1β	1.99 m		2.06 m		1.99 m	
2		88.8		88.7		88.8
3	5.70 m	79.9	5.74 d (6.0)	79.9	5.68 d (9.7)	80.0
4	3.95 m	44.4	3.51 m	44.4	3.95 m	44.4
5	5.83 m	72.2	5.78 br s	72.1	5.82 br s	72.3
6		146.4		146.4		146.4
7	4.53 s	67.2	4.32 br s	67.8	4.53 d (8.8)	67.3
8	5.27 s	72.1	5.17 s	71.9	5.26 s	71.9
9	5.11 s	82.2	4.86 s	80.8	5.10 s	82.2
10		40.8		40.4		40.7
11	6.13 d (16.0)	134.6	5.86 d (16.0)	134.6	6.14 d (15.9)	134.6
12	5.70 m	131.5	5.62 dd (16.0, 9.6)	131.2	5.71 dd (16.0, 9.5)	131.4
13	2.75 m	38.1	2.60 m	37.7	2.76 m	38.1
14	5.13 s	79.6	5.08 s	79.6	5.13 s	79.5
15		84.2		83.9		84.2
16	1.46 s	24.5	1.50 s	23.2	1.47 s	24.4
17	4.83 s; 4.41 s	108.6	4.92 s; 4.52 s	109.2	4.82 s; 4.42 s	108.4
18	1.06 s	27.3	1.01 s	26.9	1.07 s	27.4
19	1.42 s	23.6	1.35 s	23.7	1.45 s	23.6
20	1.14 d (7.0)	23.3	1.13 d (7.1)	23.3	1.15 d (7.1)	23.2
2-OAc	2.25 s	171.6	2.13 s	170.7	2.25 s	171.5
		22.5		22.3		22.5
3-OBz	8.08 d (7.3)	165.6	8.11 m	165.4	8.08 m	165.5
	7.42 m	129.8	7.45 m	129.8	7.41 m	129.7
	7.57 m	129.7	7.58 t (7.4)	129.9	7.57 m	128.4
		128.4		128.5		123.2
		133.4		133.4		133.4
5-OAc	2.07 s	168.0	2.01 s	168.1	2.08 s	167.9
		21.1		21.0		21.0
8-OAc ^3^/8-OPr ^4, 5^	2.03 s	168.9	2.27 m	172.3	2.47 m; 2.35 m	172.3
		20.7	1.06 t (7.5)	27.4	1.09 t (7.6)	27.4
				9.1		9.1
9-ONic ^3, 5^/9-OAc ^4^	9.41 s	165.0	2.17 s	170.9	9.41 m	164.9
	8.42 d (8.0)	151.4		21.1	8.42 dt (8.0, 2.0)	151.4
	7.42 m	126.3			7.44 m	133.4
	8.79 br d	137.4			8.80 dd (4.9, 1.8)	137.3
		123.3				126.4
		153.5				153.7
14-OAc	2.12 s	170.7	2.11 s	170.8	2.12 s	170.6
		20.5		20.5		20.5

^a^ Recorded in CDCl_3_ at 500 MHz (^1^H) and 125 MHz (^13^C). s, singlet; d, doublet; t, triplet; dd, doublet of doublets; dt, doublet of triplets; br s, broad singlet; m, multiplet; br d, broad doublet. Superscripts ^3, 4, 5^ represent Compound **3**, Compound **4**, Compound **5** respectively.

## Data Availability

The data supporting the findings of this study are available from the corresponding authors upon reasonable request.

## Supplementary Materials

The following supporting information can be downloaded at https://www.mdpi.com/article/10.3390/molecules31173024/s1, Figures S1–S10: Spectral data (^1^H NMR, ^13^C NMR, ^1^H–^1^H COSY, HSQC, HMBC, ROESY, (+)-HR-ESI-MS, CD, UV, IR) for compound **1** (Euphjatrophane M); Figures S11–S20: Corresponding spectral data (^1^H NMR, ^13^C NMR, ^1^H–^1^H COSY, HSQC, HMBC, ROESY, (+)-HR-ESI-MS, CD, UV, IR) for compound **2** (Euphjatrophane N); Figures S21–S30: Corresponding spectral data (^1^H NMR, ^13^C NMR, ^1^H–^1^H COSY, HSQC, HMBC, ROESY, (+)-HR-ESI-MS, CD, UV, IR) for compound **3** (Euphjatrophane O); Figures S31–S40: Corresponding spectral data (^1^H NMR, ^13^C NMR, ^1^H–^1^H COSY, HSQC, HMBC, ROESY, (+)-HR-ESI-MS, CD, UV, IR) for compound **4** (Euphjatrophane P); Figures S41–S50: Corresponding spectral data (^1^H NMR, ^13^C NMR, ^1^H–^1^H COSY, HSQC, HMBC, ROESY, (+)-HR-ESI-MS, CD, UV, IR) for compound **5** (Euphjatrophane Q).

## Abbreviations

The following abbreviations are used in this manuscript:

NMR	Nuclear magnetic resonance
HR-ESI-MS	High-resolution electrospray ionization mass spectrometry
UV	Ultraviolet–visible spectroscopy
CD	Circular dichroism
IR	Infrared spectroscopy
HSQC	Heteronuclear single-quantum coherence
HMBC	Heteronuclear multiple-bond correlation
ROESY	Rotating-frame Overhauser effect spectroscopy
^1^H–^1^H COSY	^1^H–^1^H correlation spectroscopy
AD	Alzheimer’s disease
Baf A1	Bafilomycin A1
TFEB	Transcription factor EB
