# Supplementary material for: Jatrophane Diterpenoids from Euphorbia peplus with LysoTracker-Associated Cellular Activity
Source: Molecules. 2026 Aug 28;31(17):3024. doi: 10.3390/molecules31173024 (PMC13567447; doi:10.3390/molecules31173024)
Supplement: Supplementary file 1 [file molecules-31-03024-s001.zip › molecules-4504814-supplementary.pdf]

Supporting Information

Jatrophane Diterpenoids from *Euphorbia peplus* with LysoTracker-Associated Cellular Activity

Mingrui Yuan [1,2], Shipeng Guan [3], Bowen Ouyang [1,2], Zifei Xu [1], Peng Wang [4], Qing Zhao [3], Chonglin Yang [5], Xiaojiang Hao [1], Qiuyuan Yin [5,*] and Yingtong Di [1,*]

1 State Key Laboratory of Phytochemistry and Natural Medicines, Kunming Institute of Botany, Chinese Academy of Sciences, Kunming 650201, China
2 University of Chinese Academy of Sciences, Beijing 100049, China
3 School of Chinese Materia Medica, Yunnan University of Traditional Chinese Medicine, Kunming 650500, China
4 Science and Technology Information Center, Kunming Institute of Botany, Chinese Academy of Sciences, Kunming 650201, China
5 Yunnan Key Laboratory of Natural Medicinal Chemistry, Yunnan University, Kunming 650091, China

* Correspondence: qiuyuan_yin@ynu.edu.cn (Q.Y.); diyt@mail.kib.ac.cn (Y.D.)

List of Contents

Figure S1. ^{1}H NMR spectrum of Euphjatrophane M 5

Figure S2. ^{13}C NMR spectrum of Euphjatrophane M 5

Figure S3. ^{1}H-^{1}H COSY spectrum of Euphjatrophane M 6

Figure S4. HSQC spectrum of Euphjatrophane M 6

Figure S5. HMBC spectrum of Euphjatrophane M 7

Figure S6. ROESY spectrum of Euphjatrophane M 7

Figure S7. (+)-HRESIMS spectrum of Euphjatrophane M 8

Figure S8. CD spectrum of Euphjatrophane M 9

Figure S9. UV spectrum of Euphjatrophane M 9

Figure S10. IR spectrum of Euphjatrophane M 10

Figure S11. ^{1}H NMR spectrum of Euphjatrophane N 10

Figure S12. ^{13}C NMR spectrum of Euphjatrophane N 11

Figure S13. ^{1}H-^{1}H COSY spectrum of Euphjatrophane N 11

Figure S14. HSQC spectrum of Euphjatrophane N 12

Figure S15. HMBC spectrum of Euphjatrophane N 12

Figure S16. ROESY spectrum of Euphjatrophane N 13

Figure S17. (+)-HRESIMS spectrum of Euphjatrophane N 14

Figure S18. CD spectrum of Euphjatrophane N 15

Figure S19. UV spectrum of Euphjatrophane N 15

Figure S20. IR spectrum of Euphjatrophane N 16

Figure S21. ^{1}H NMR spectrum of Euphjatrophane O 16

Figure S22. ^{13}C NMR spectrum of Euphjatrophane O 17

Figure S23. ^{1}H-^{1}H COSY spectrum of Euphjatrophane O 17

Figure S24. HSQC spectrum of Euphjatrophane O 18

Figure S25. HMBC spectrum of Euphjatrophane O 18

Figure S26. ROESY spectrum of Euphjatrophane O 19

Figure S27. (+)-HRESIMS spectrum of Euphjatrophane O 20

Figure S28. CD spectrum of Euphjatrophane O 21

Figure S29. UV spectrum of Euphjatrophane O 21

Figure S30. IR spectrum of Euphjatrophane O 22

Figure S31. ^{1}H NMR spectrum of Euphjatrophane P 22

Figure S32. ^{13}C NMR spectrum of Euphjatrophane P 23

Figure S33. ^{1}H-^{1}H COSY spectrum of Euphjatrophane P 23

Figure S34. HSQC spectrum of Euphjatrophane P 24

Figure S35. HMBC spectrum of Euphjatrophane P 24

Figure S36. ROESY spectrum of Euphjatrophane P 25

Figure S37. (+)-HRESIMS spectrum of Euphjatrophane P 26

Figure S38. CD spectrum of Euphjatrophane P 27

Figure S39. UV spectrum of Euphjatrophane P 27

Figure S40. IR spectrum of Euphjatrophane P 28

Figure S41. ^{1}H NMR spectrum of Euphjatrophane Q 28

Figure S42. ^{13}C NMR spectrum of Euphjatrophane Q 29

Figure S43. ^{1}H-^{1}H COSY spectrum of Euphjatrophane Q 29

Figure S44. HSQC spectrum of Euphjatrophane Q **30**

Figure S45. HMBC spectrum of Euphjatrophane Q **30**

Figure S46. ROESY spectrum of Euphjatrophane Q **31**

Figure S47. (+)-HRESIMS spectrum of Euphjatrophane Q **32**

Figure S48. CD spectrum of Euphjatrophane Q **33**

Figure S49. UV spectrum of Euphjatrophane Q **33**

Figure S50. IR spectrum of Euphjatrophane Q **34**

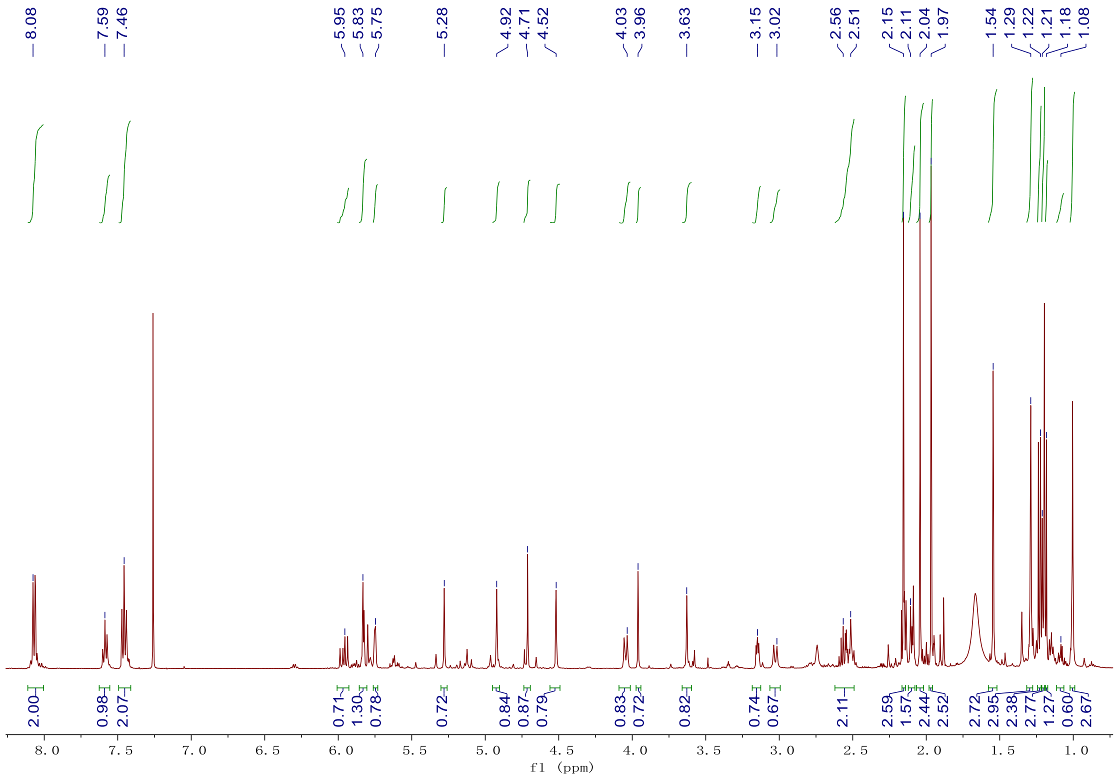

Figure S1. ^{1}H-NMR spectrum of Euphjatrophane M

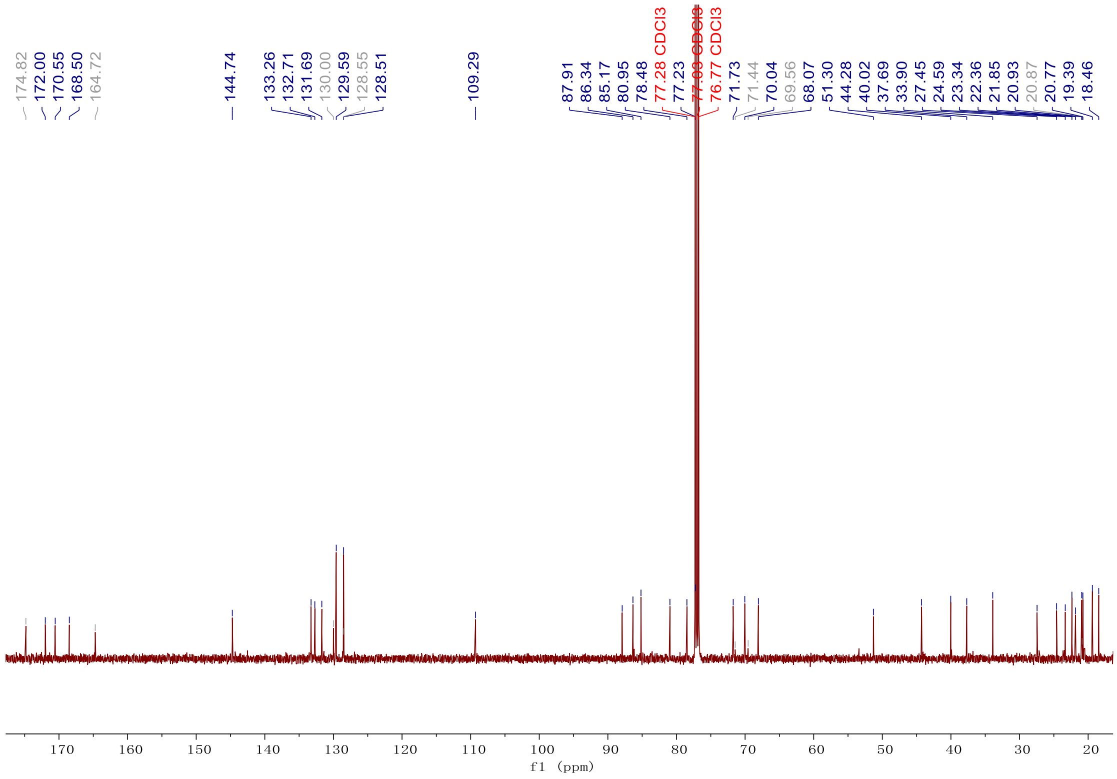

Figure S2. ^{13}C-NMR spectrum of Euphjatrophane M

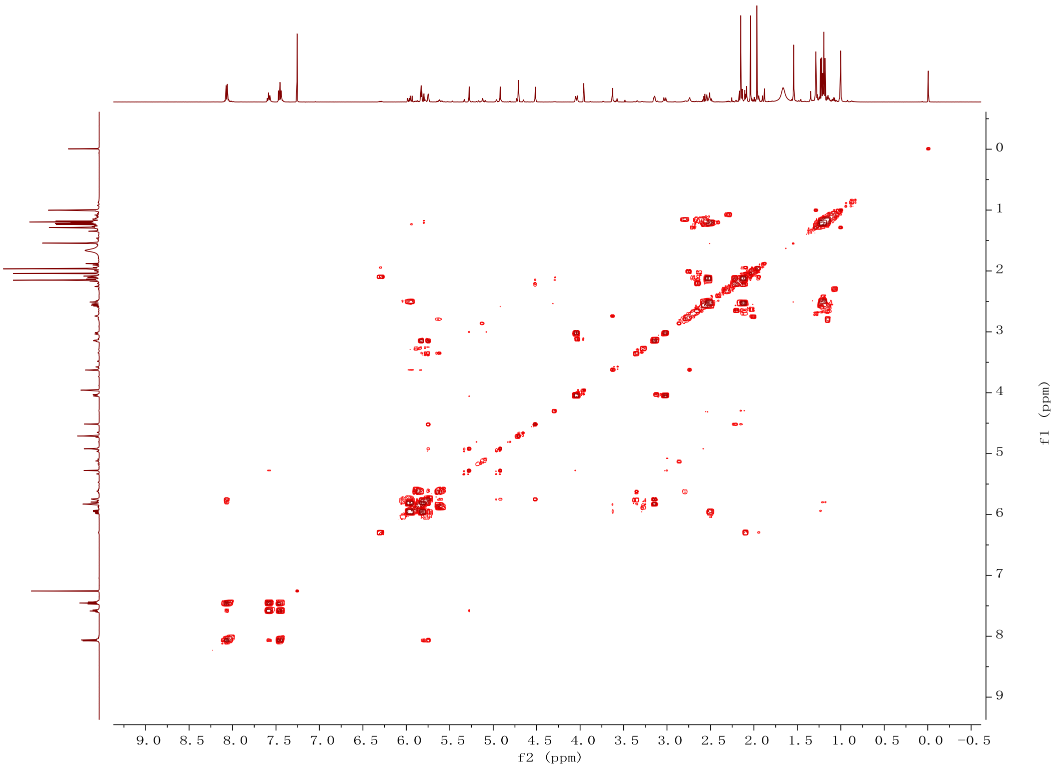

Figure S3. COSY spectrum of Euphjatrophane M

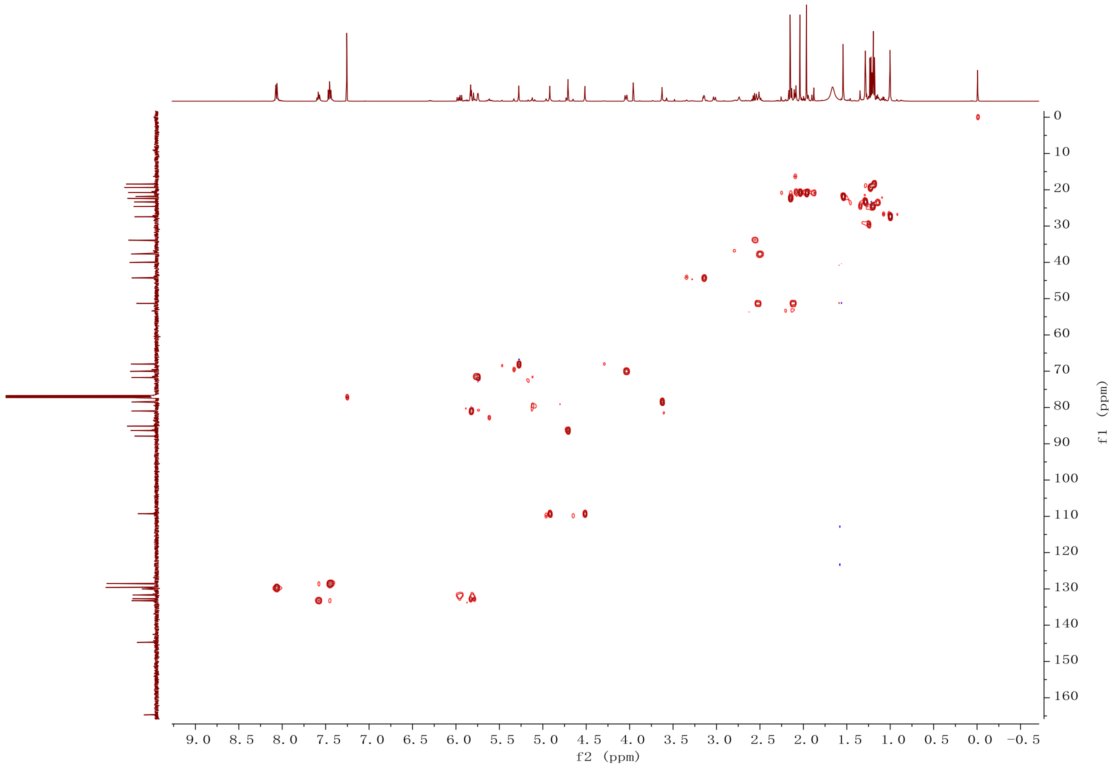

Figure S4. HSQC spectrum of Euphjatrophane M

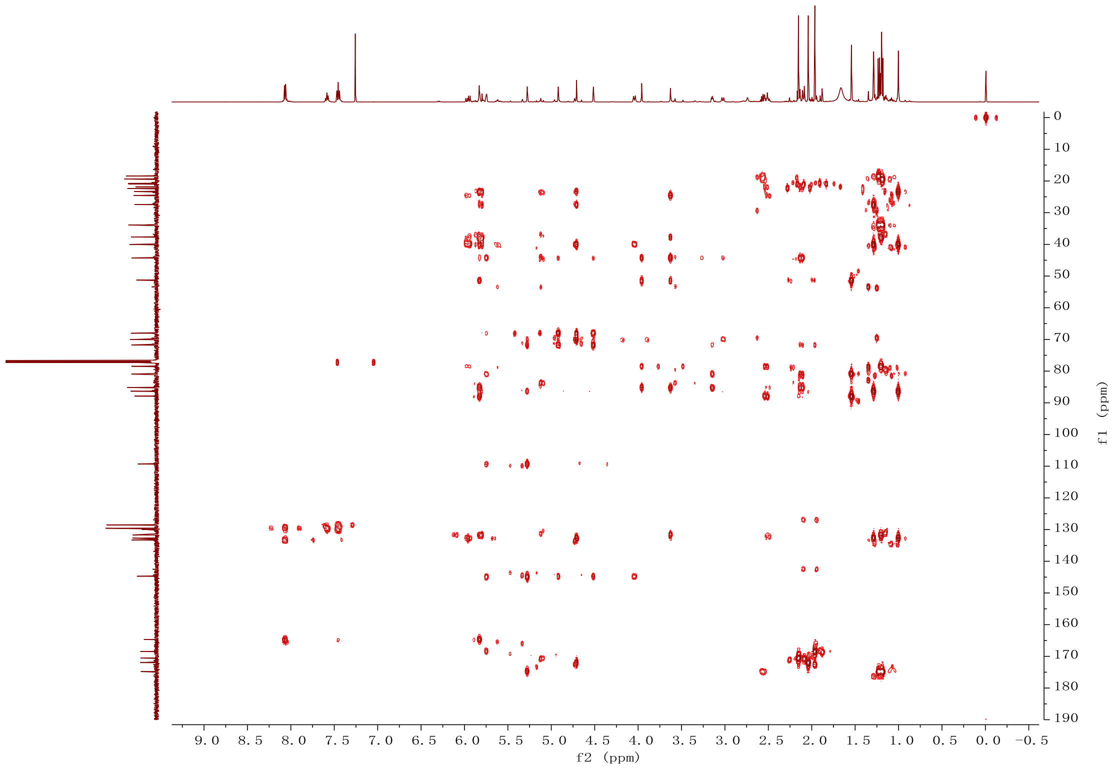

Figure S5. HMBC spectrum of Euphjatrophane M

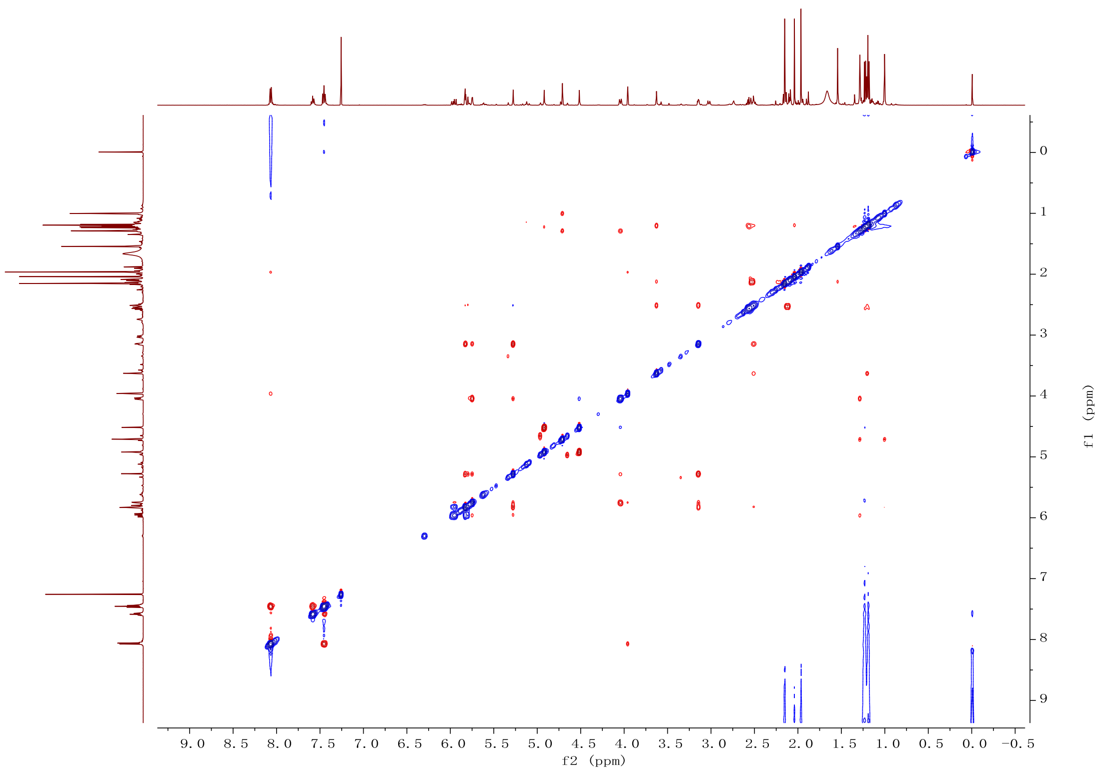

Figure S6. ROESY spectrum of Euphjatrophane M

Qualitative Analysis Report

**Data Filename**	h-gsp-29.d	**Sample Name**	h-gsp-29
**Sample Type**	Sample	**Position**	P1-A4
**Instrument Name**	Instrument 1	**User Name**	
**Acq Method**	s.m	**Acquired Time**	2/10/2023 11:19:32 AM
**IRM Calibration Status**	Success	**DA Method**	PCDL.m
**Comment**			

**Sample Group**		**Info.**
**Acquisition SW Version**	6200 series TOF/6500 series Q-TOF B.05.01 (B5125.2)	

User Spectra

Fragmentor Voltage	Collision Energy	Ionization Mode
135	0	ESI

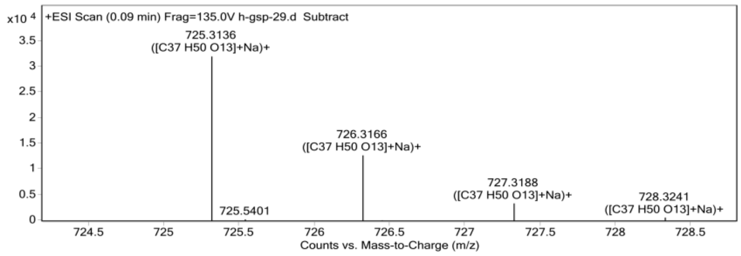

Peak List

m/z	z	Abund	Formula	Ion
678.348	1	9160.23		
683.3029	1	6303.06		
720.3589	1	38454.22		
721.3618	1	14963.53		
722.365	1	4338.81		
725.3136	1	32046.44	C37 H50 O13	(M+Na)+
726.3166	1	12852.45	C37 H50 O13	(M+Na)+
732.3585	1	3753.04		
748.3558	1	6798.08		
753.3081	1	5159.68		

Formula Calculator Element Limits

Element	Min	Max
C	3	60
H	0	150
O	0	20
N	0	3

Formula Calculator Results

Formula	CalculatedMass	CalculatedMz	Mz	Diff. (mDa)	Diff. (ppm)	DBE
C37 H50 O13	702.3251	725.3144	725.3136	0.80	1.10	13.0000

--- End Of Report ---

Figure S7. HR-ESI-MS spectrum of Euphjatrophane M

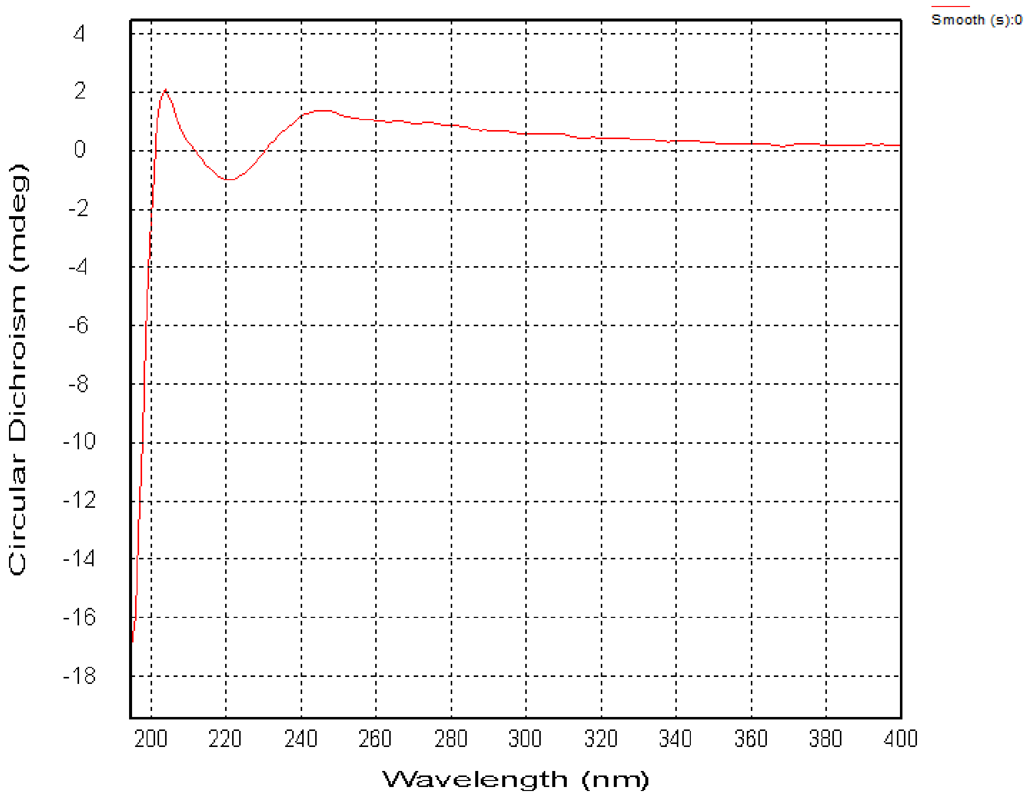

Figure S8. CD spectrum of Euphjatrophane M

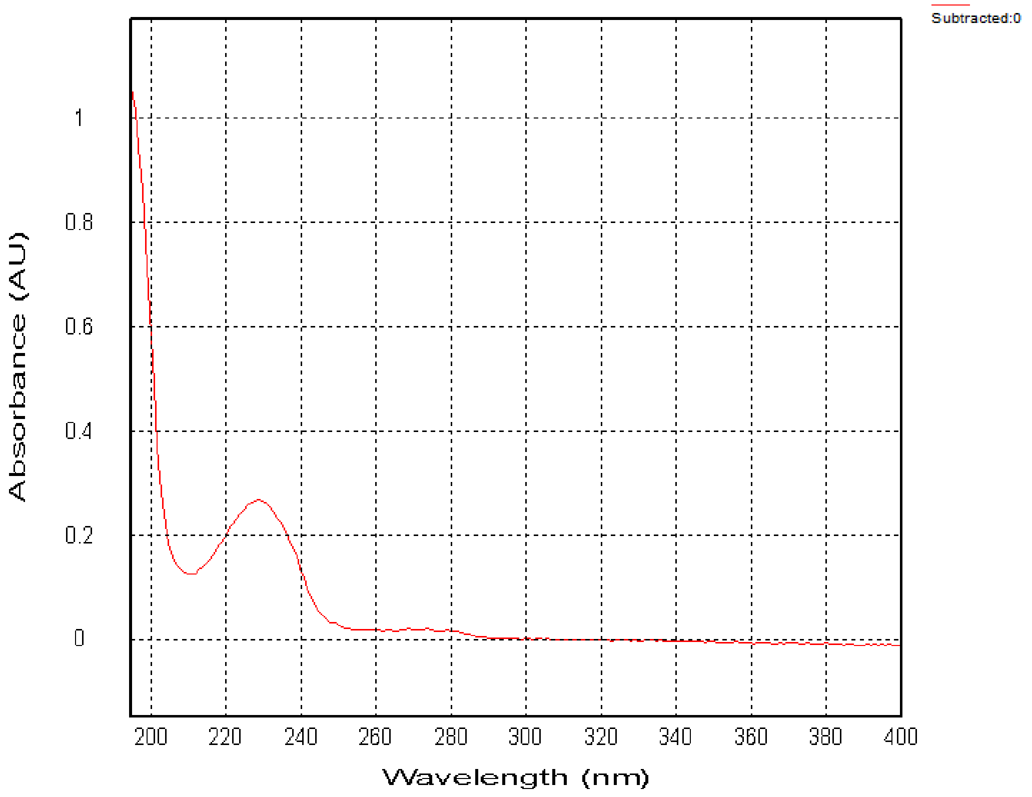

Figure S9. UV spectrum of Euphjatrophane M

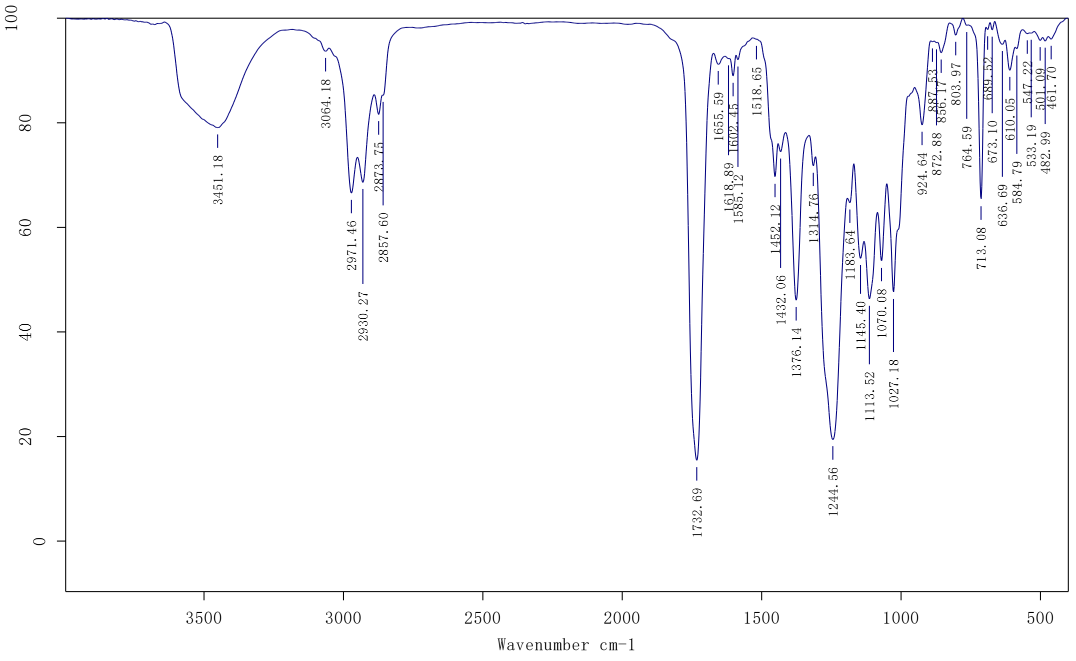

Sample Name: hgsp29
Sample Form: KBr
Path of File: E:\data
Date of Measurement: 2022/9/9

Resolution: 4
Aperture Setting: 6 mm
Number of Background Scans: 16
Number of Sample Scans: 16

Beamsplitter Setting: KBr
Source Setting: MIR
Instrument Type: BRUKER VERTEX 70
Soft Version: OPUS8.1

Figure S10. IR spectrum of Euphjatrophane M

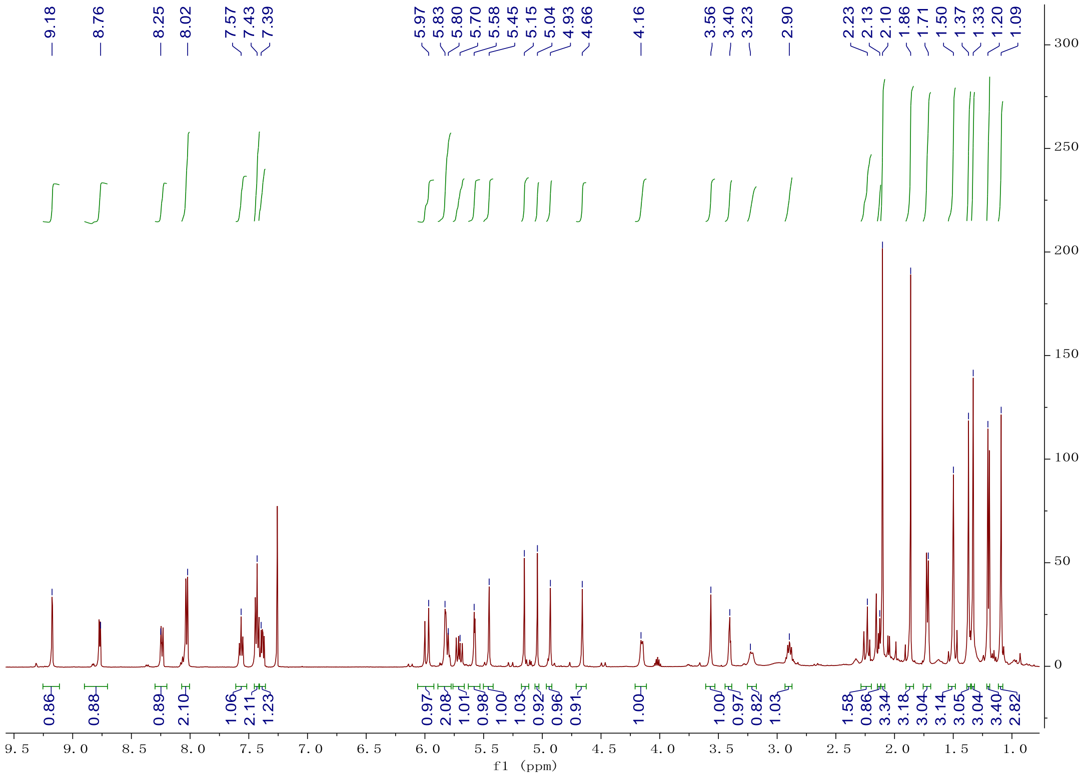

Figure S11. ^{1}H-NMR spectrum of Euphjatrophane N

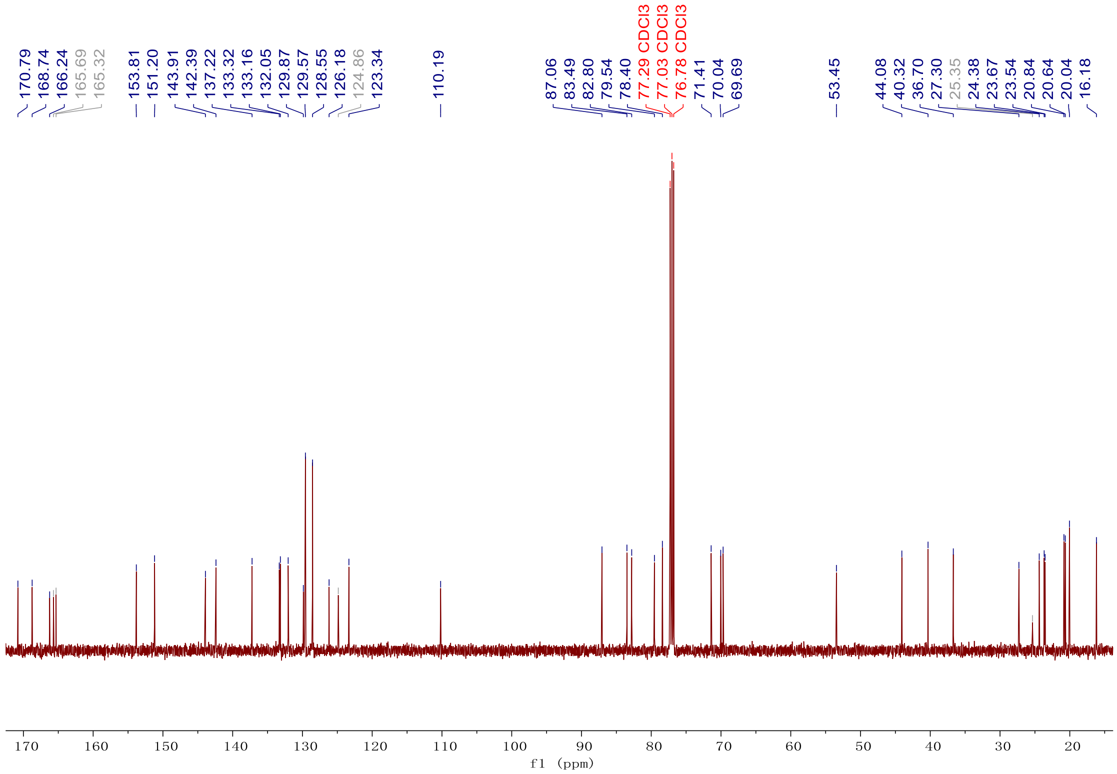

Figure S12. ^{13}C-NMR spectrum of Euphjatrophane N

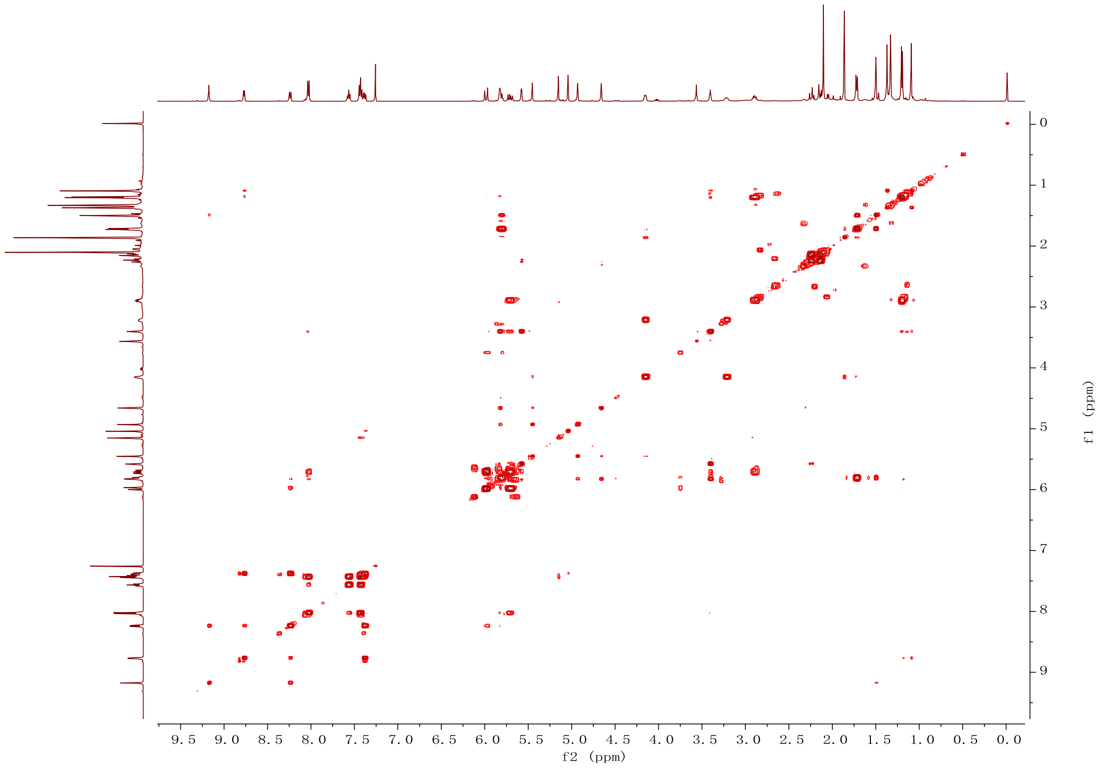

Figure S13. COSY spectrum of Euphjatrophane N

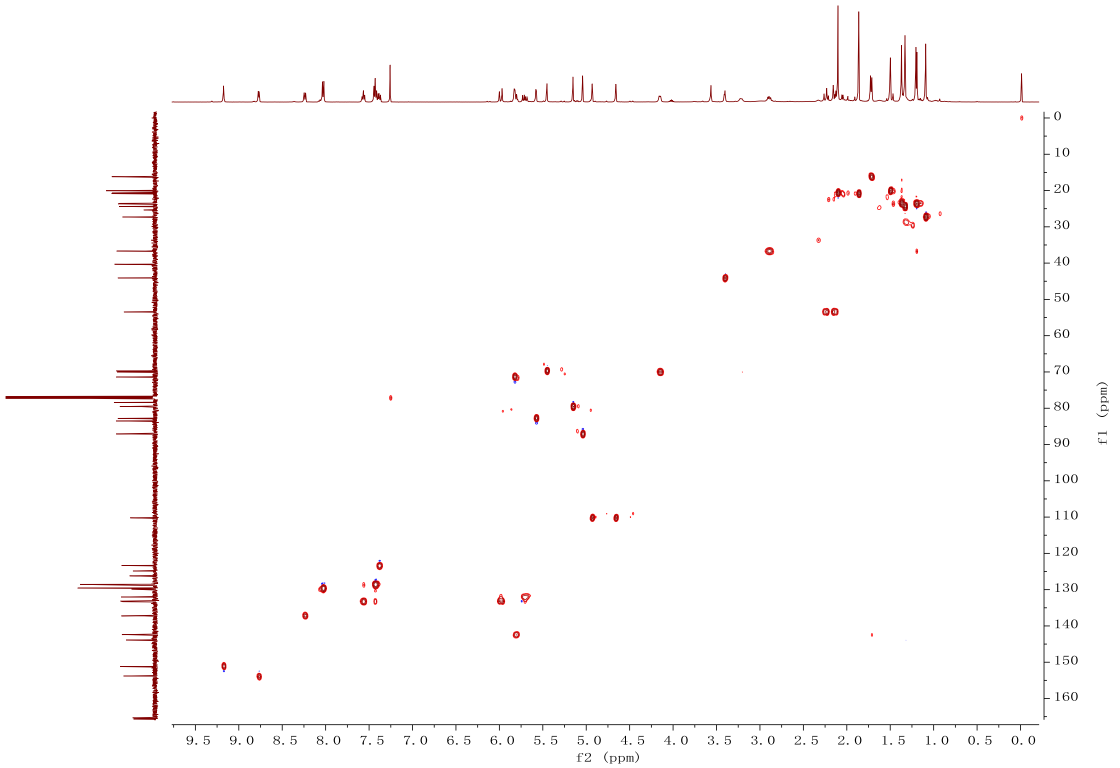

Figure S14. HSQC spectrum of Euphjatrophane N

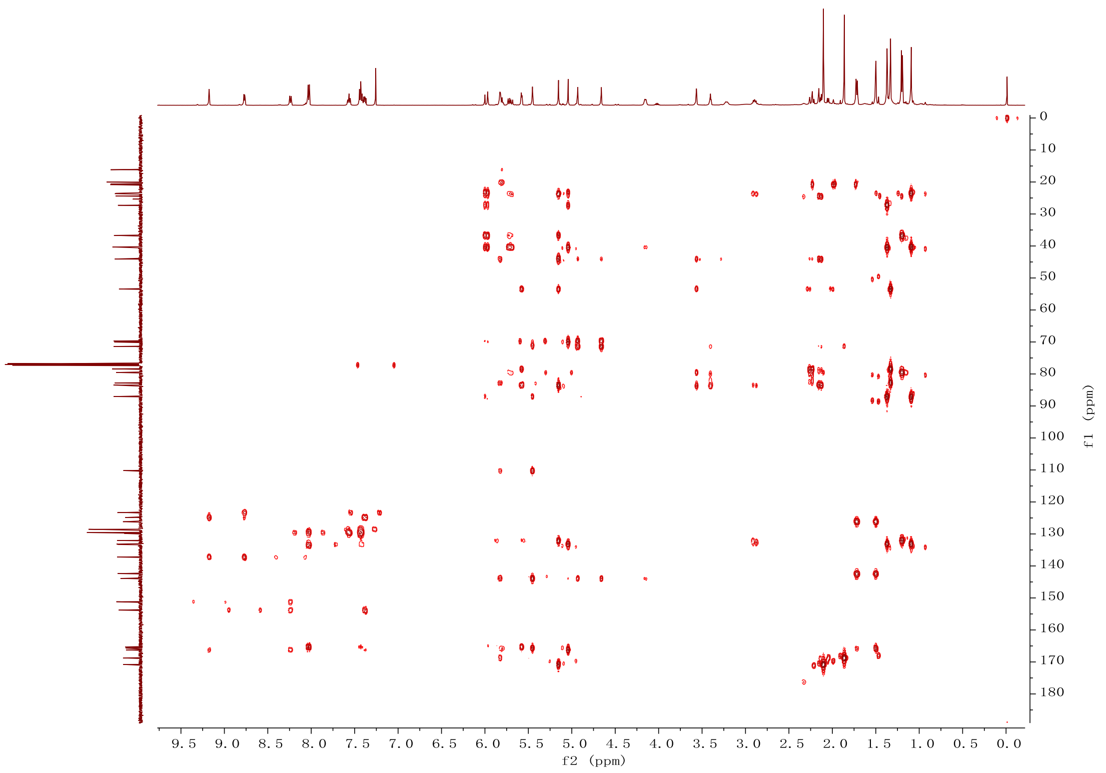

Figure S15. HMBC spectrum of Euphjatrophane N

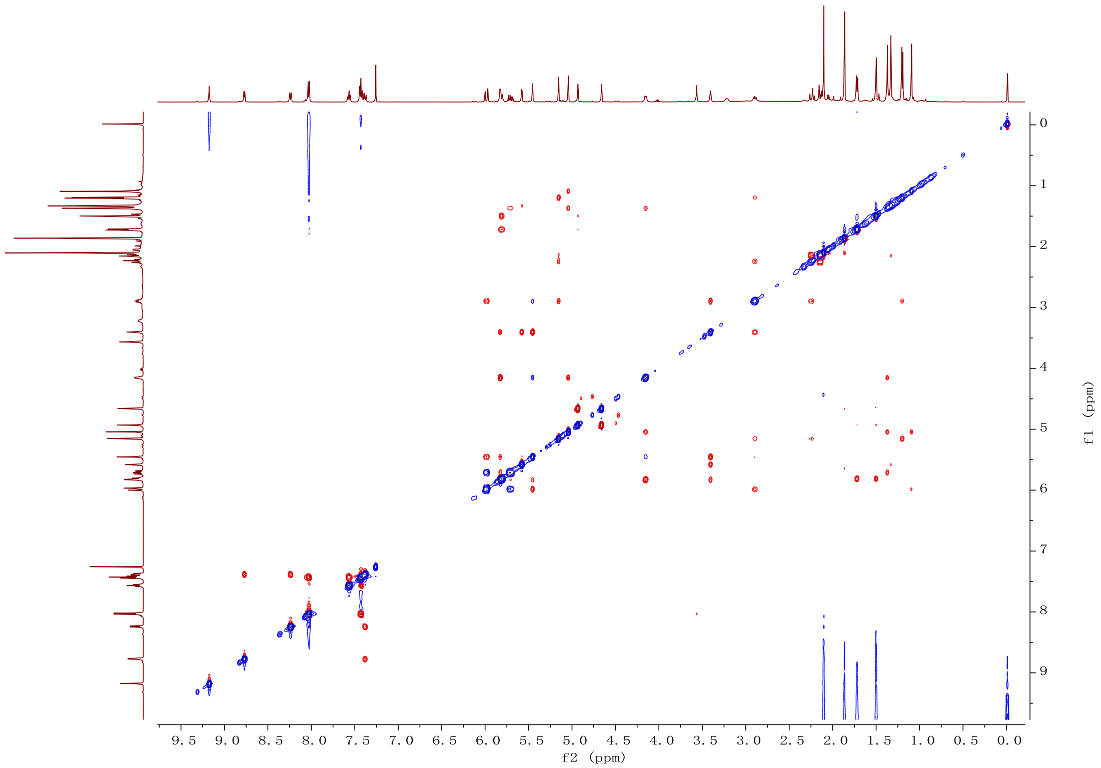

Figure S16. ROESY spectrum of Euphjatrophane N

Qualitative Analysis Report

**Data Filename**	h-gsp-40.d	**Sample Name**	h-gsp-40
**Sample Type**	Sample	**Position**	P1-A7
**Instrument Name**	Instrument 1	**User Name**	
**Acq Method**	s.m	**Acquired Time**	2/10/2023 11:22:55 AM
**IRM Calibration Status**	Success	**DA Method**	PCDL.m
**Comment**			

**Sample Group**		**Info.**
**Acquisition SW Version**	6200 series TOF/6500 series Q-TOF B.05.01 (B5125.2)	

User Spectra

Fragmentor Voltage	Collision Energy	Ionization Mode
135	0	ESI

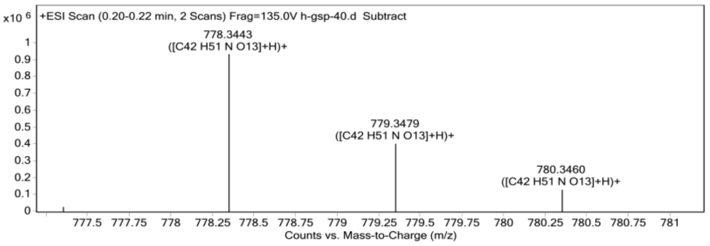

Peak List

m/z	z	Abund	Formula	Ion
776.349	1	76902.48		
778.3443	1	935863.94	C42 H51 N O13	(M+H)+
779.3479	1	406594.41	C42 H51 N O13	(M+H)+
780.346	1	133792.52	C42 H51 N O13	(M+H)+
781.3146	1	80527.71		
800.3258	1	111331.3		
1555.6806	1	169895.91		
1556.6842	1	156749.77		
1557.6861	1	75890.97		
1577.6619	1	73320.16		

Formula Calculator Element Limits

Element	Min	Max
C	3	60
H	0	150
O	0	20
N	0	3

Formula Calculator Results

Formula	CalculatedMass	CalculatedMz	Mz	Diff. (mDa)	Diff. (ppm)	DBE
C42 H51 N O13	777.3360	778.3433	778.3443	-1.00	-1.28	18.0000

--- End Of Report ---

Figure S17. HR-ESI-MS spectrum of Euphjatrophane N

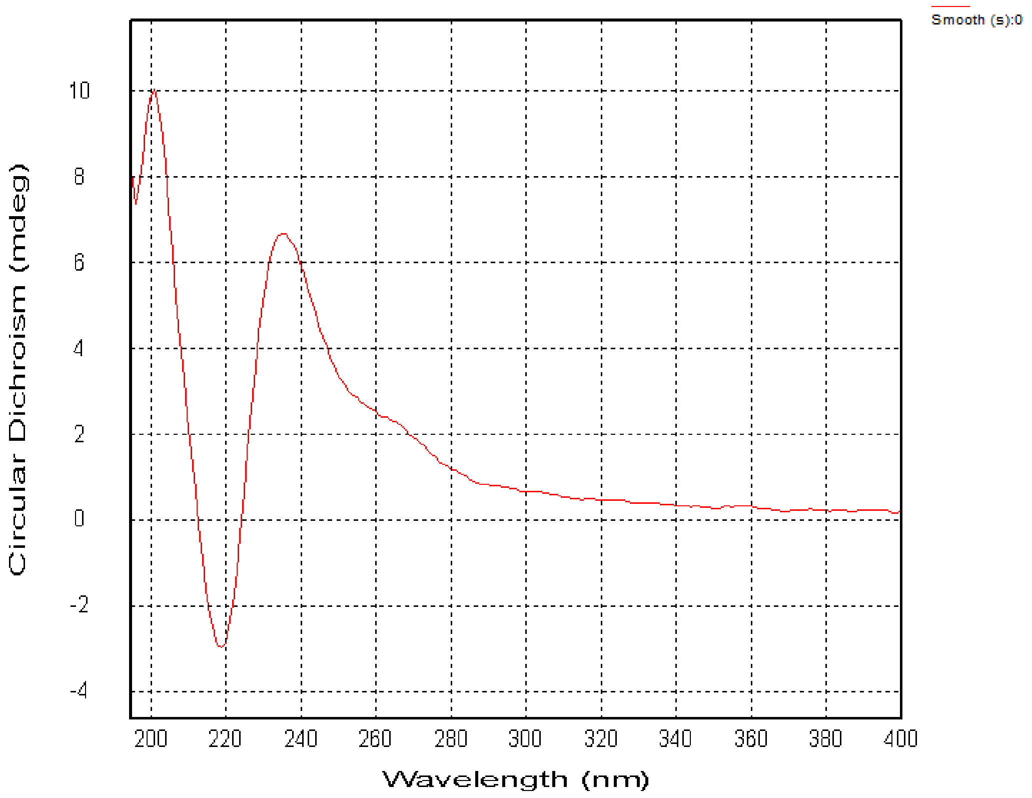

Figure S18. CD spectrum of Euphjatrophane N

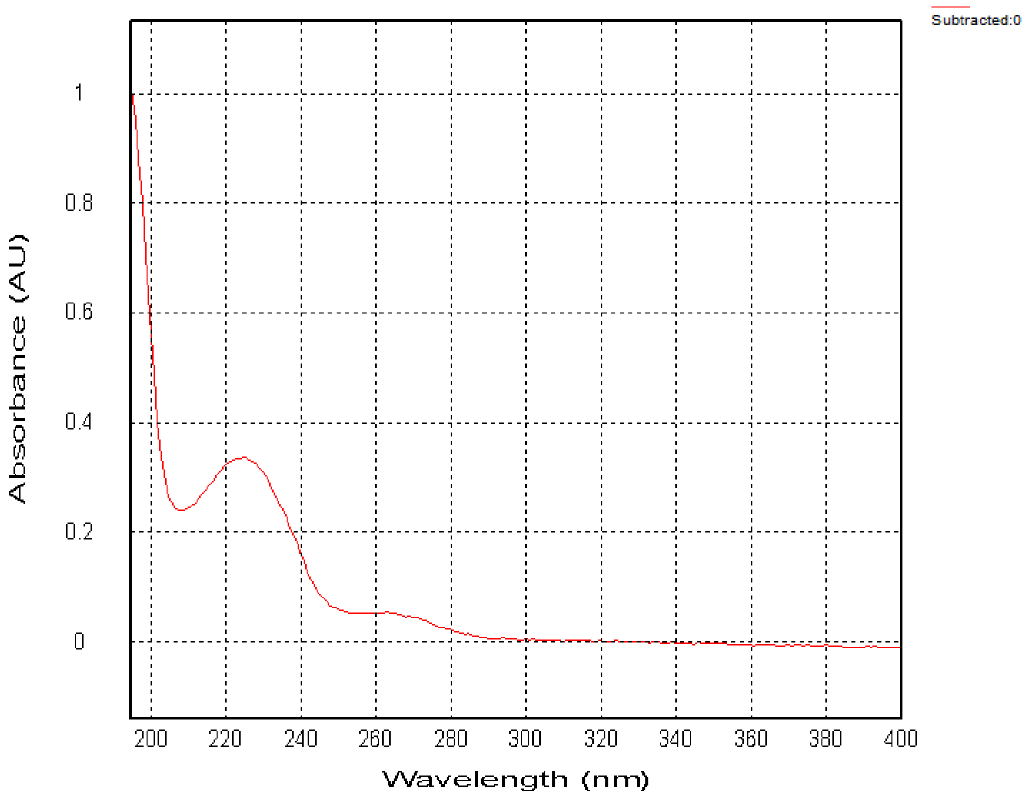

Figure S19. UV spectrum of Euphjatrophane N

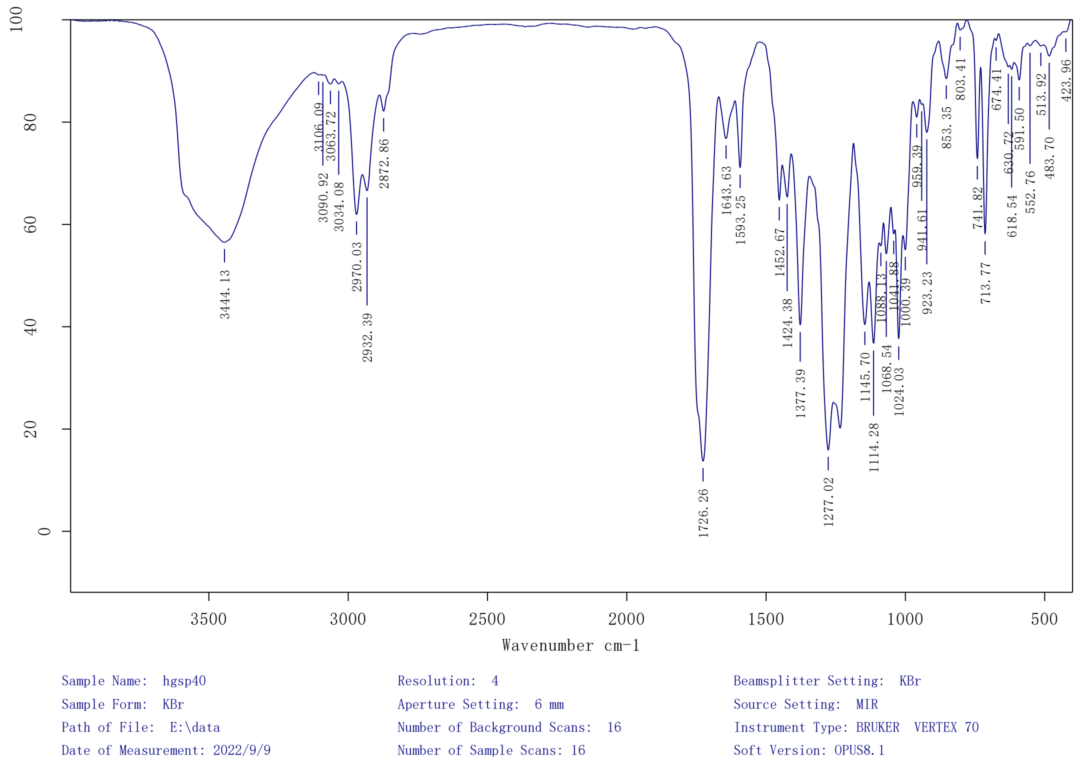

Figure S20. IR spectrum of Euphjatrophane N

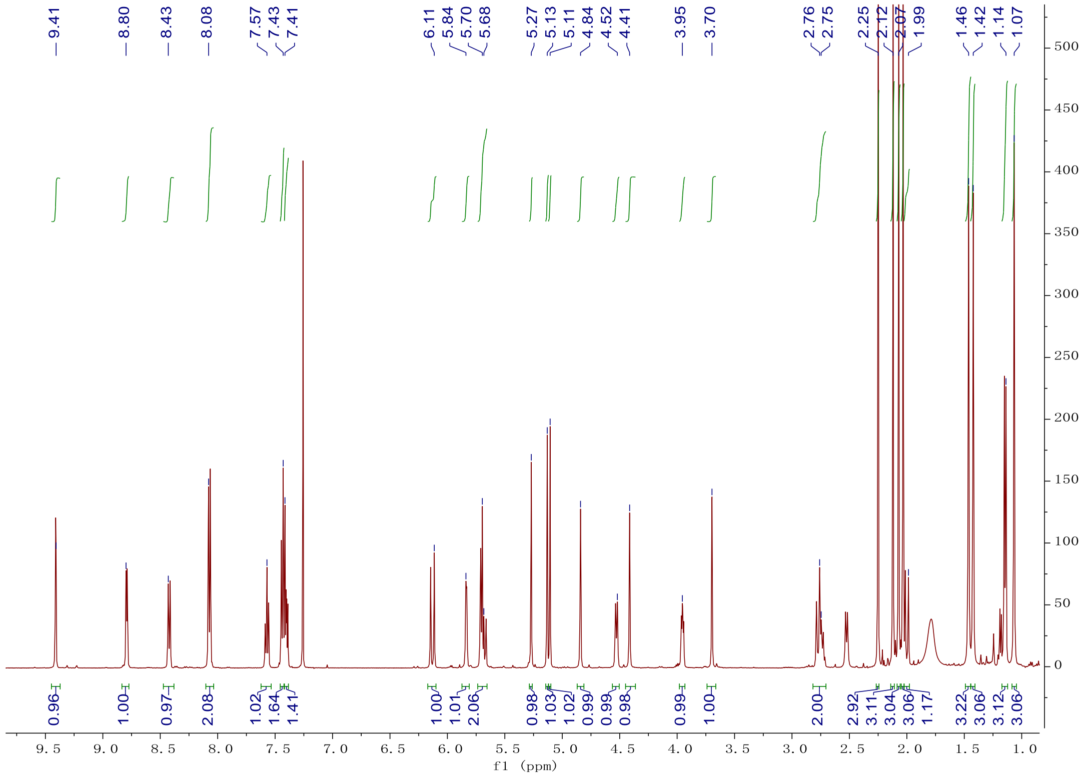

Figure S21. ^{1}H-NMR spectrum of Euphjatrophane O

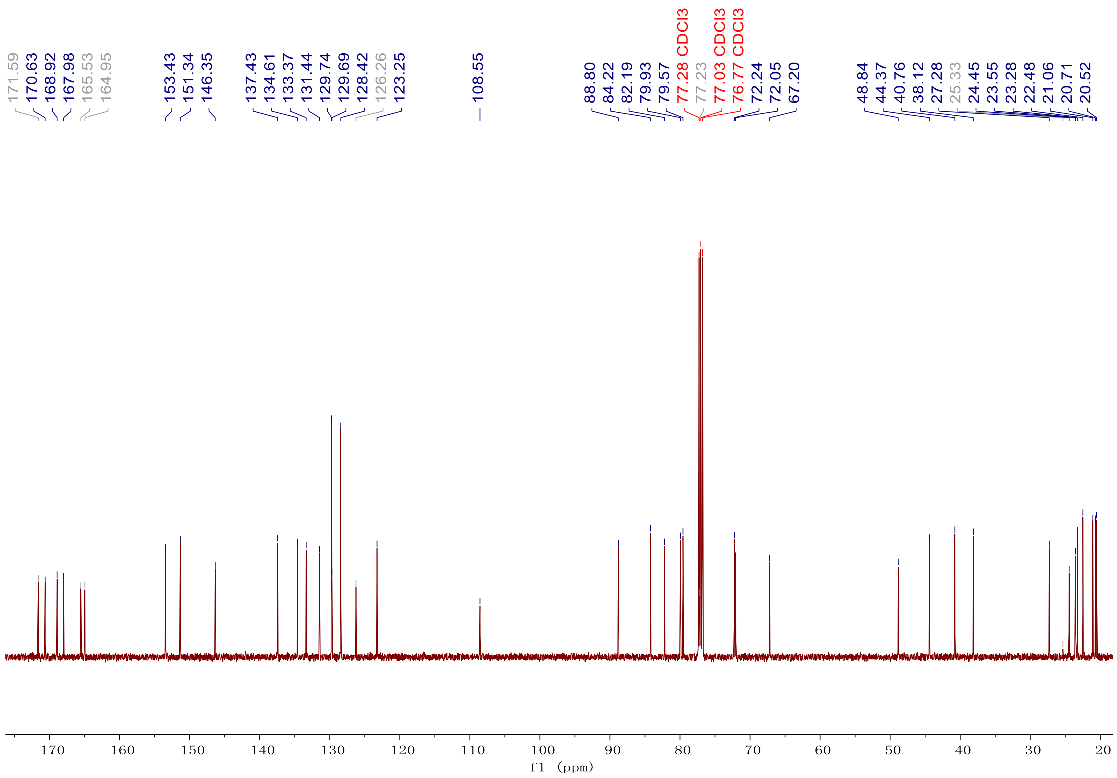

Figure S22. ^{13}C-NMR spectrum of Euphjatrophane O

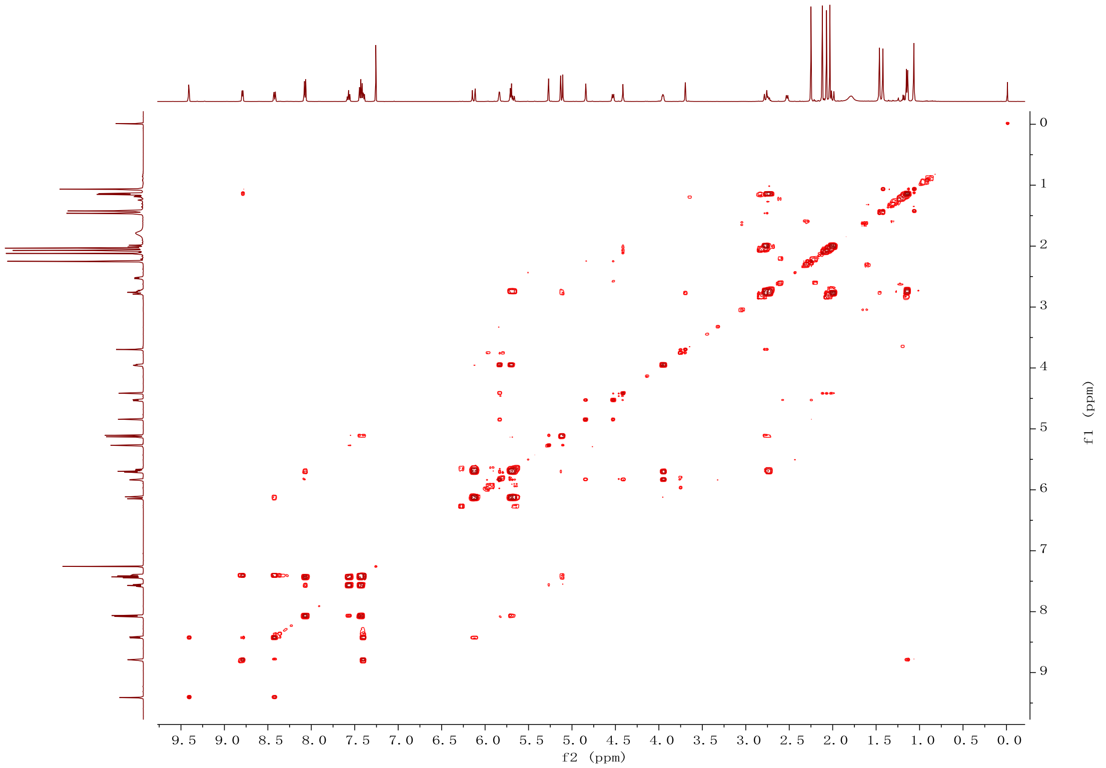

Figure S23. COSY spectrum of Euphjatrophane O

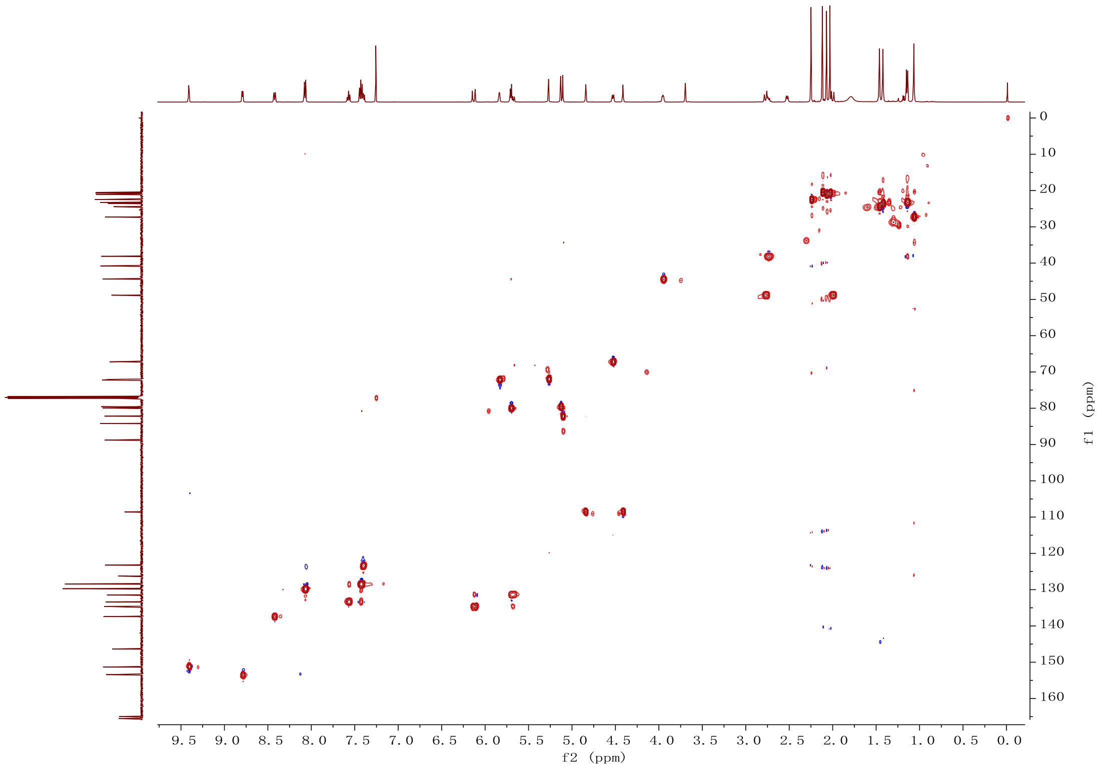

Figure S24. HSQC spectrum of Euphjatrophane O

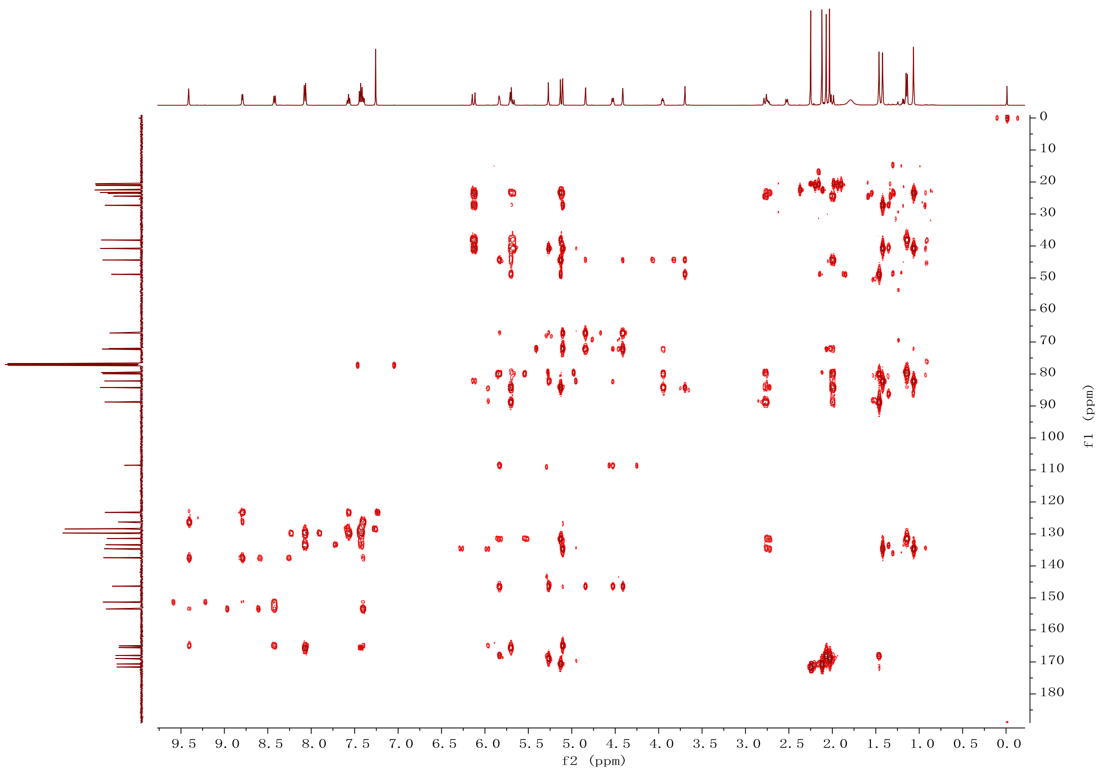

Figure S25. HMBC spectrum of Euphjatrophane O

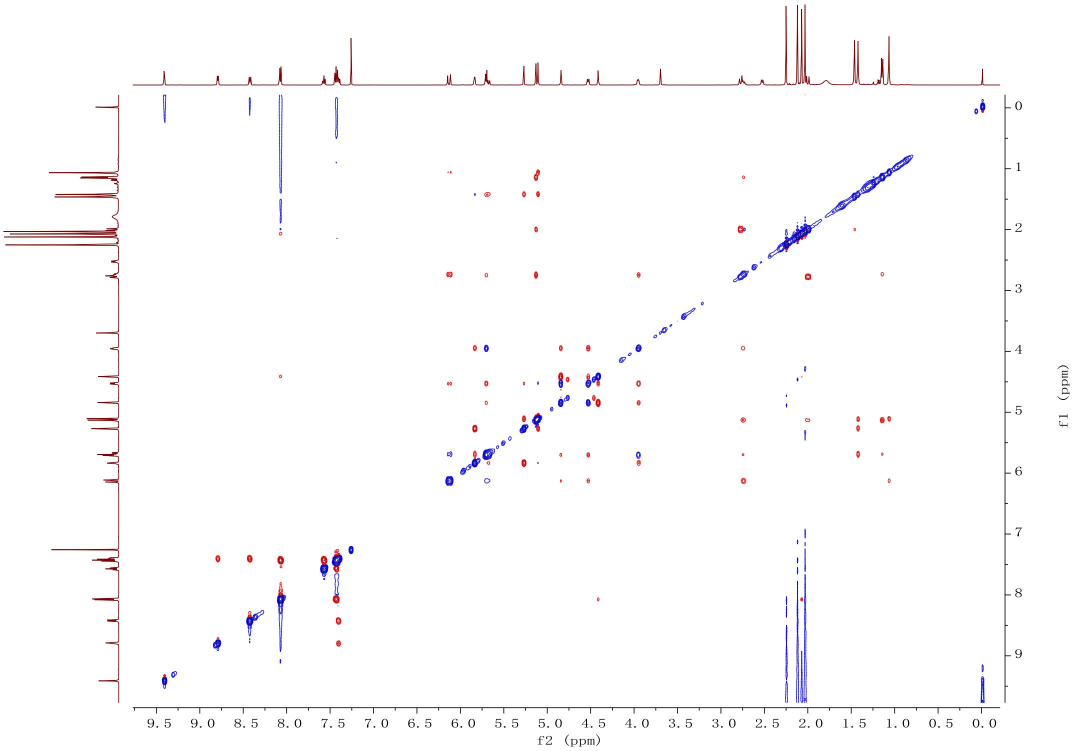

Figure S26. ROESY spectrum of Euphjatrophane O

Qualitative Analysis Report

**Data Filename**	h-gsp-39.d	**Sample Name**	h-gsp-39
**Sample Type**	Sample	**Position**	P1-A6
**Instrument Name**	Instrument 1	**User Name**	
**Acq Method**	s.m	**Acquired Time**	2/10/2023 11:21:47 AM
**IRM Calibration Status**	Success	**DA Method**	PCDL.m
**Comment**			

Sample Group **Info.**

Acquisition SW 6200 series TOF/6500 series
Version Q-TOF B.05.01 (B5125.2)

User Spectra

Fragmentor Voltage	Collision Energy	Ionization Mode
135	0	ESI

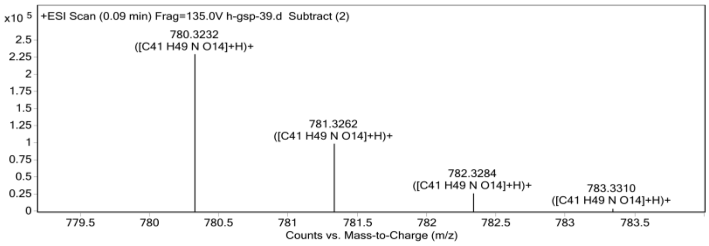

Peak List

m/z	z	Abund	Formula	Ion
780.3232	1	229925.8	C41 H49 N O14	(M+H)+
781.3262	1	99370.39	C41 H49 N O14	(M+H)+
782.3284	1	27225.87	C41 H49 N O14	(M+H)+
802.3043	1	73498.61		
803.3077	1	32122.04		
804.3106	1	10243.28		
825.3797	1	5923.43		
848.3585	1	6285.27		
1581.6199	1	12365.02		
1582.622	1	10599.53		

Formula Calculator Element Limits

Element	Min	Max
C	3	60
H	0	150
O	0	20
N	0	3

Formula Calculator Results

Formula	CalculatedMass	CalculatedMz	Mz	Diff. (mDa)	Diff. (ppm)	DBE
C41 H49 N O14	779.3153	780.3226	780.3232	-0.60	-0.77	18.0000

--- End Of Report ---

Figure S27. HR-ESI-MS spectrum of Euphjatrophane O

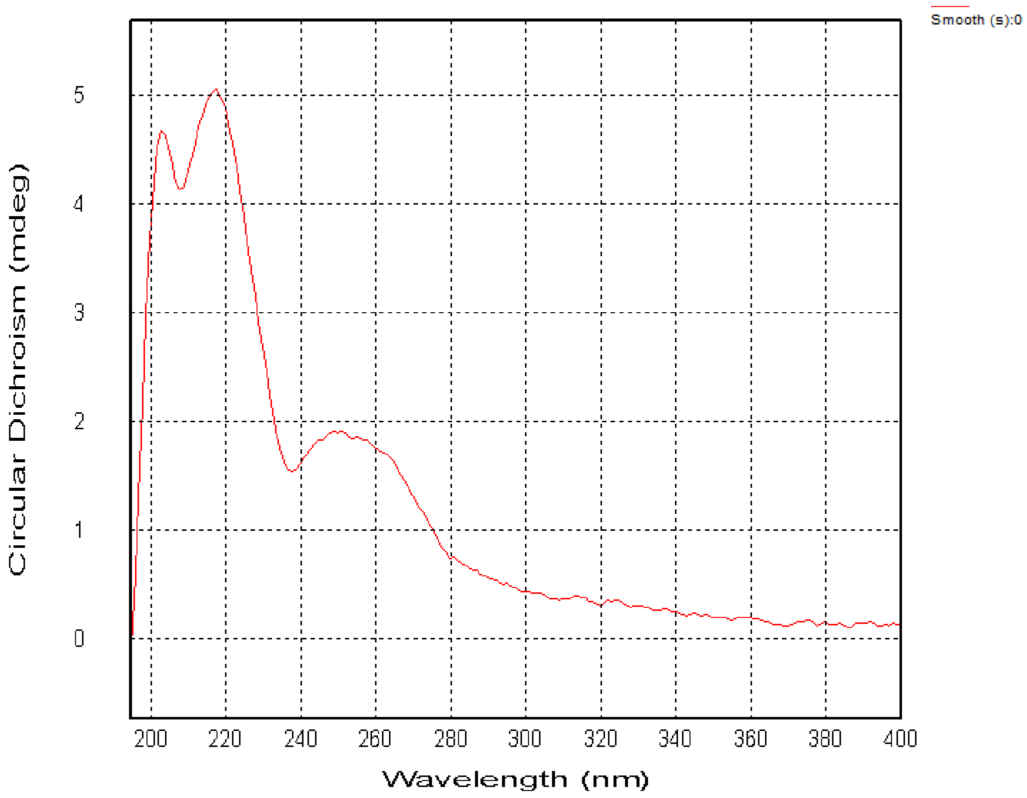

Figure S28. CD spectrum of Euphjatrophane O

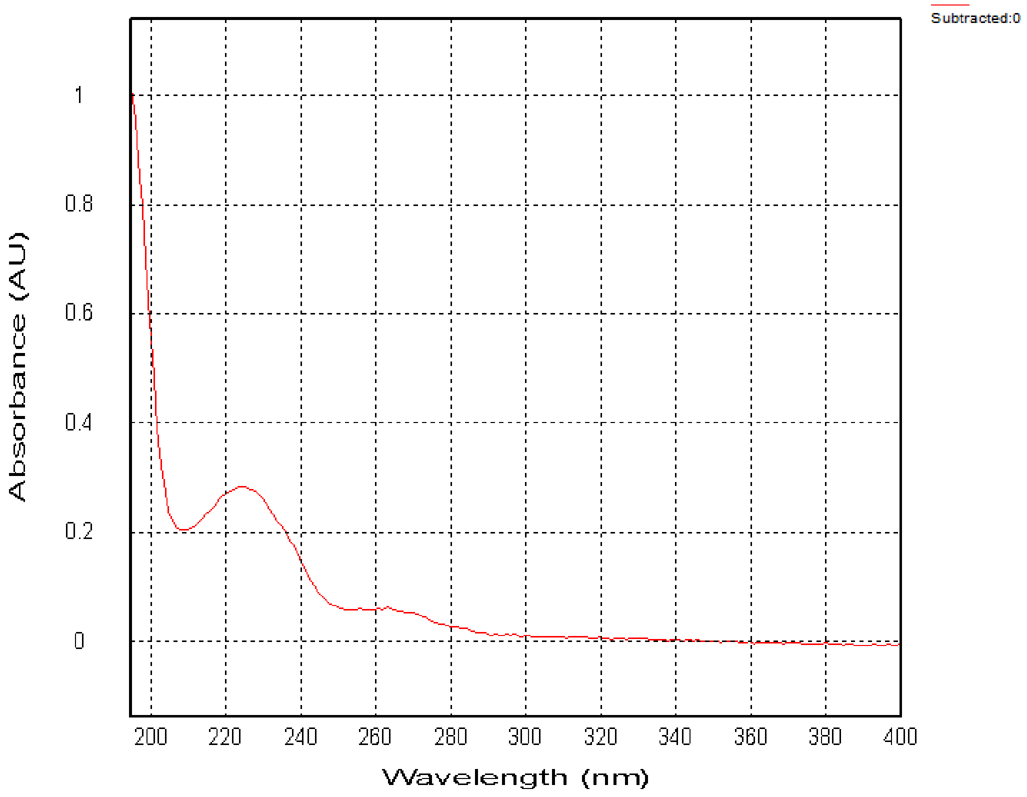

Figure S29. UV spectrum of Euphjatrophane O

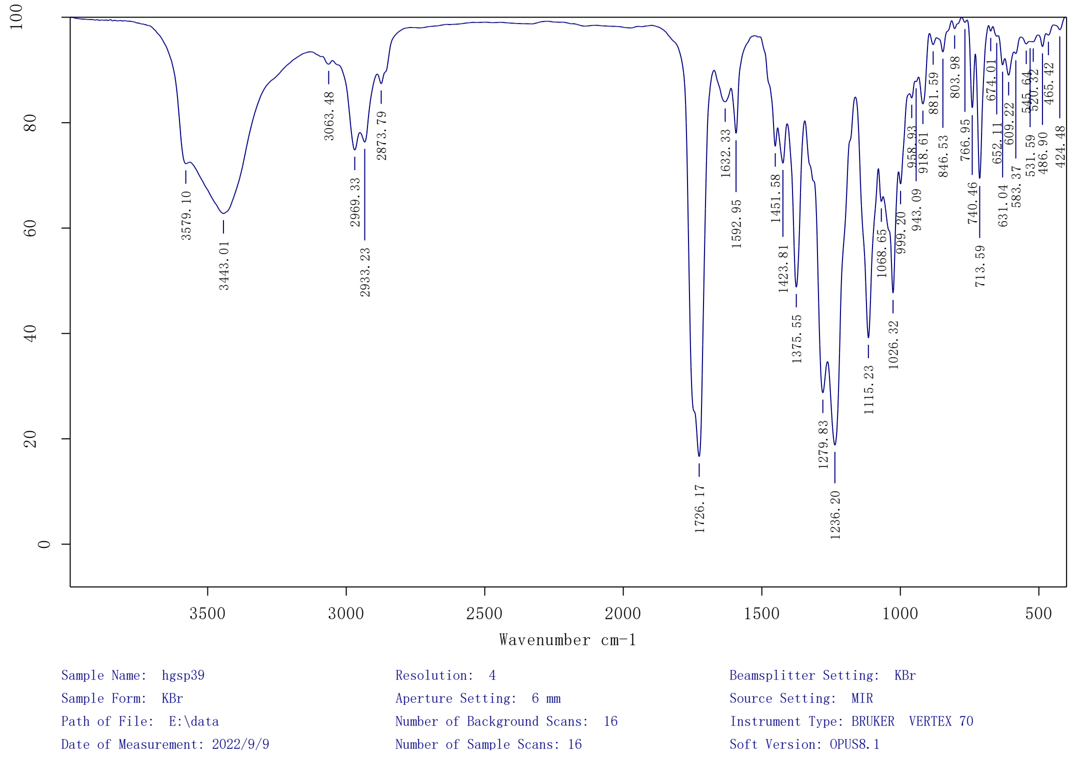

Figure S30. IR spectrum of Euphjatrophane O

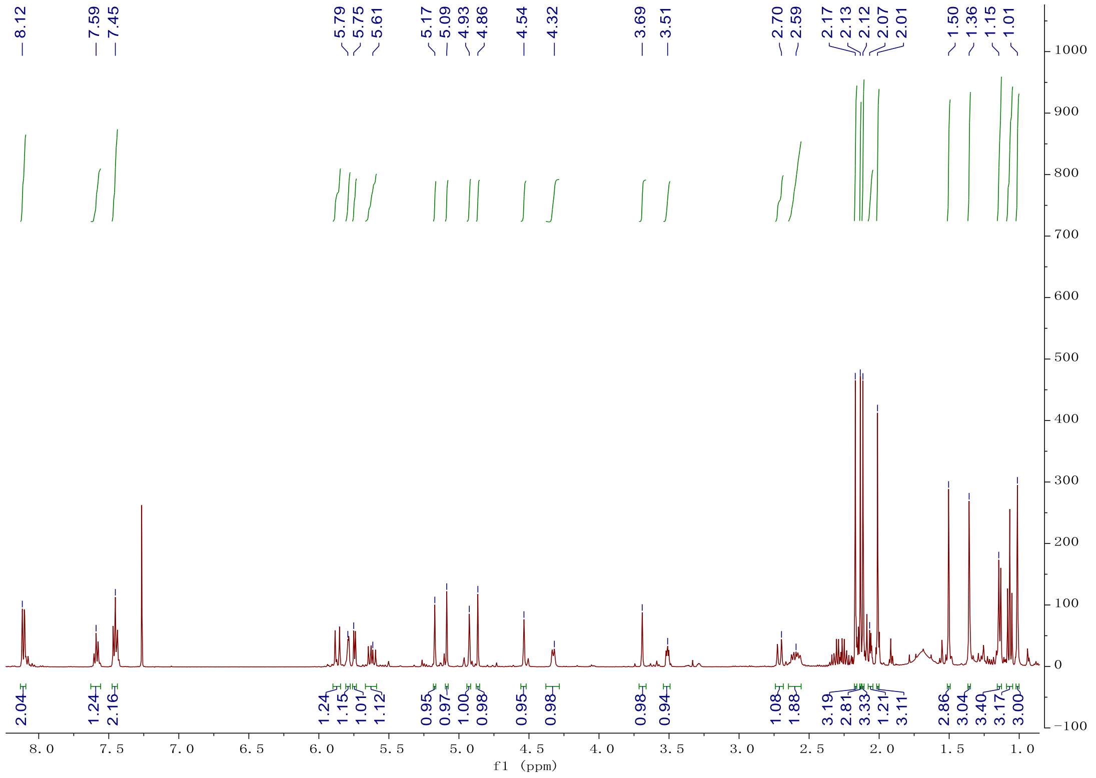

Figure S31. ^{1}H-NMR spectrum of Euphjatrophane P

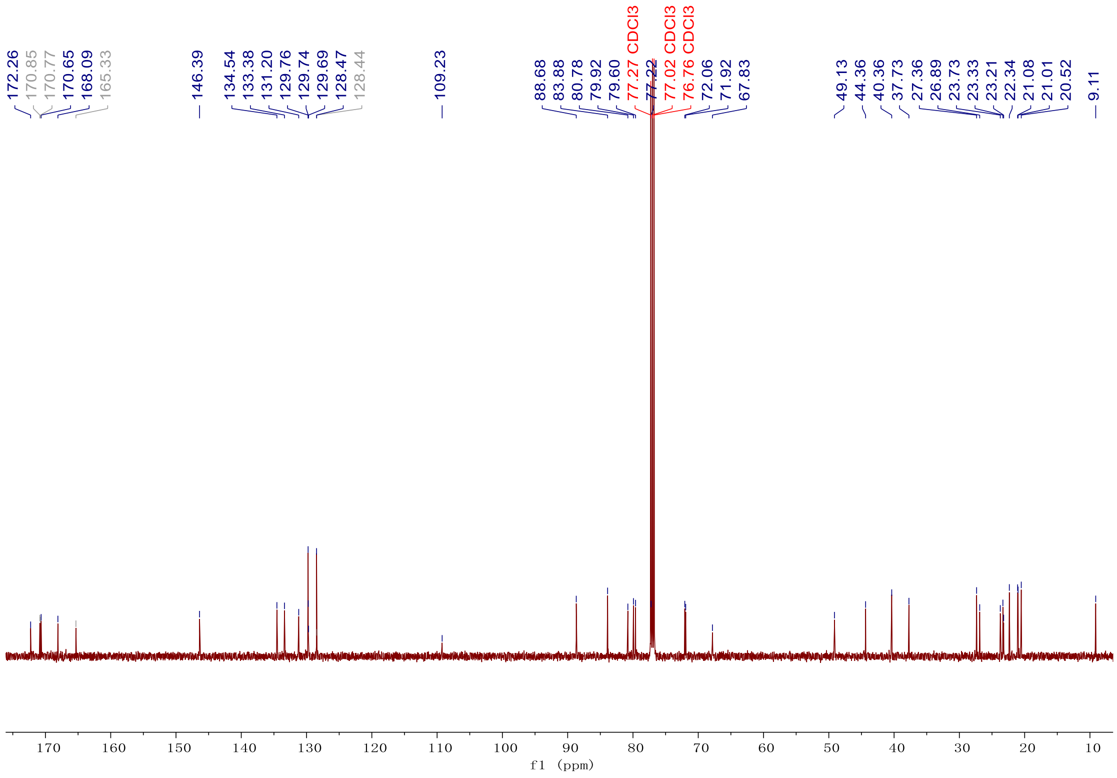

Figure S32. ^{13}C-NMR spectrum of Euphjatrophane P

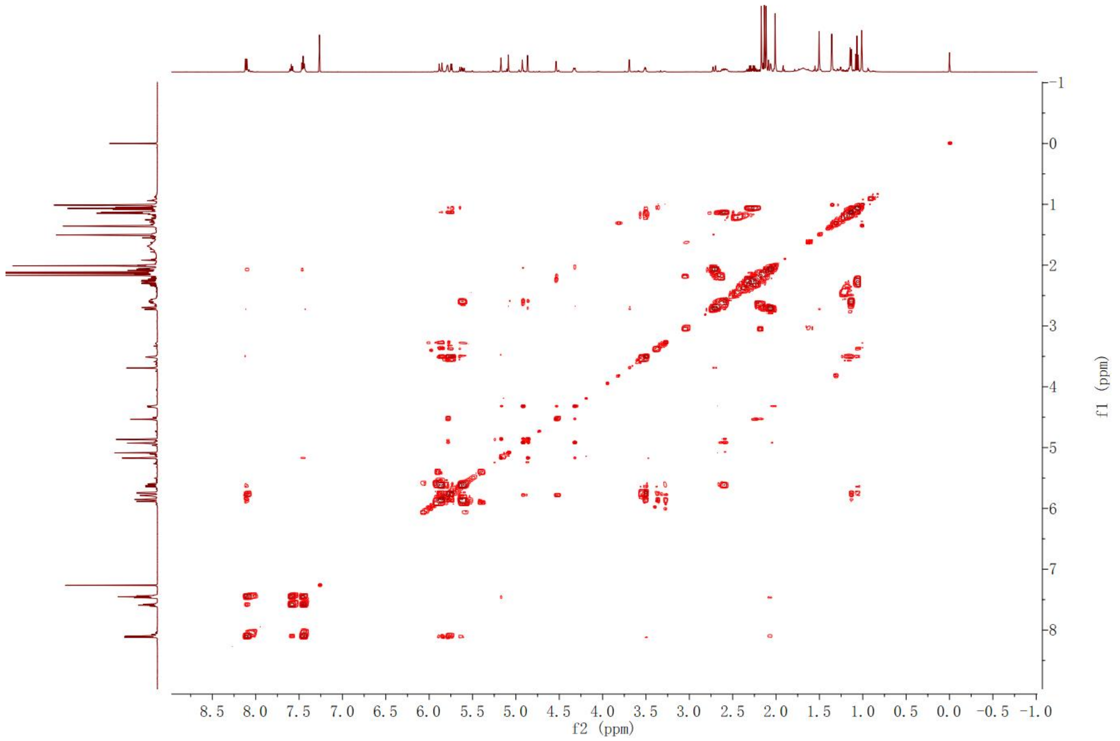

Figure S33. ^{1}H-^{1}H COSY spectrum of Euphjatrophane P

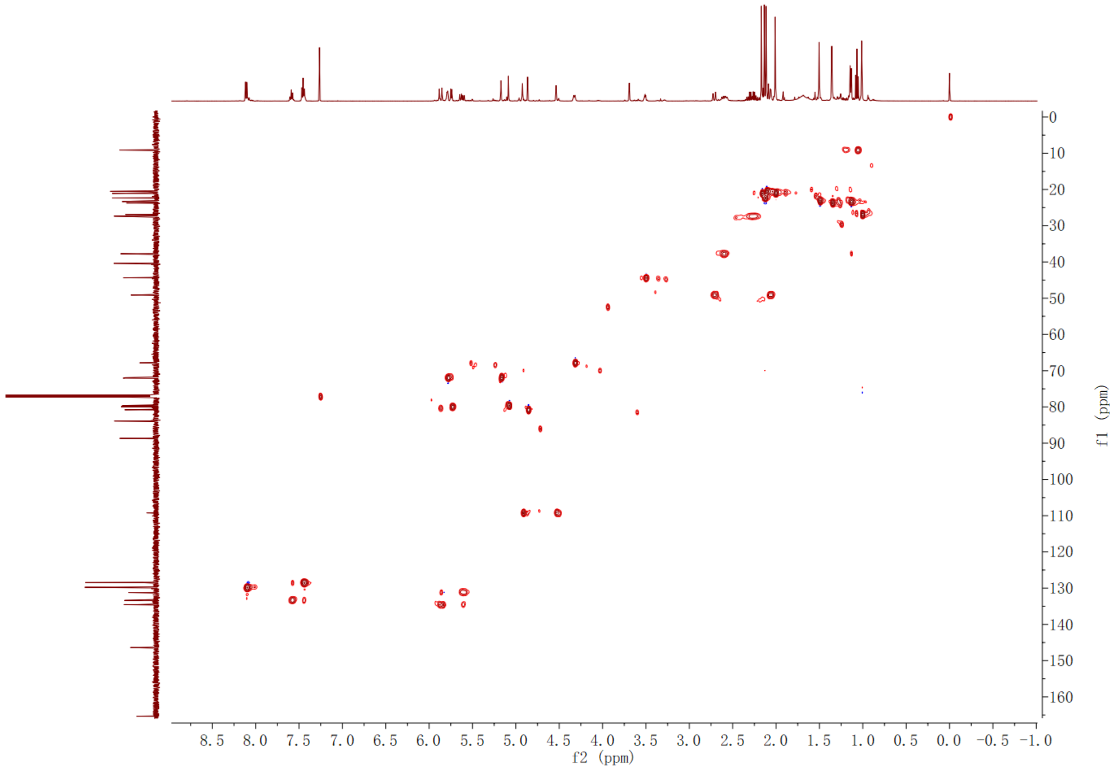

Figure S34. HSQC spectrum of Euphjatrophane P

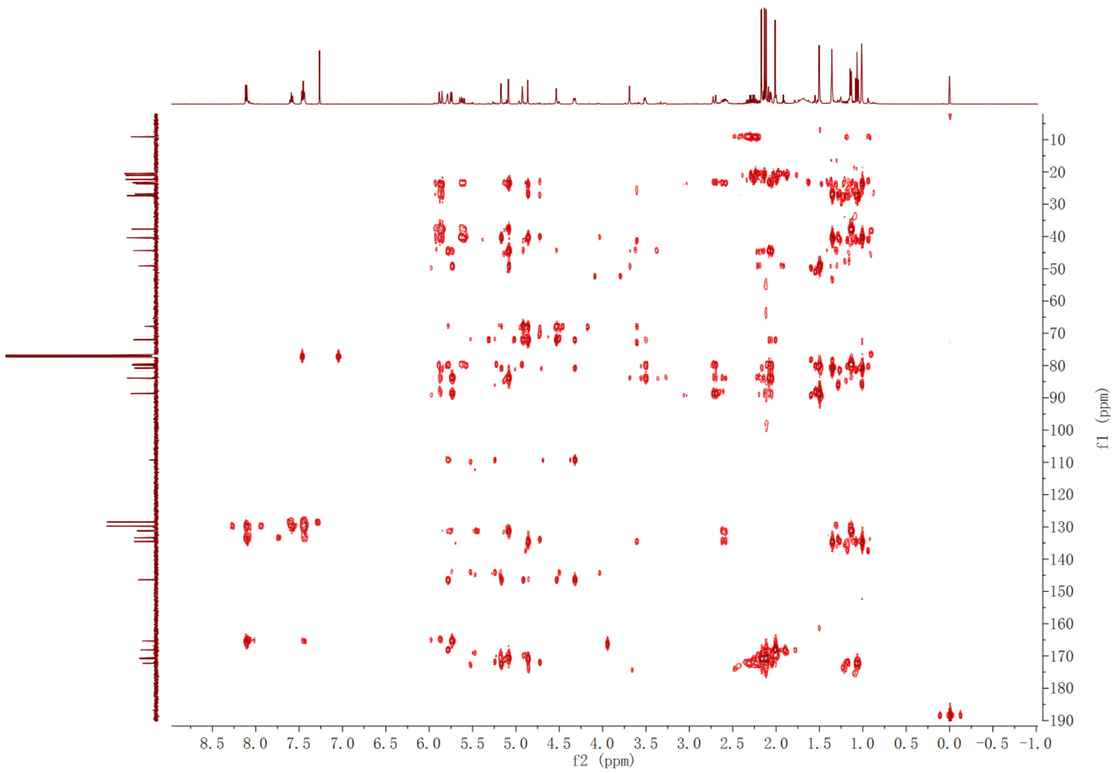

Figure S35. HMBC spectrum of Euphjatrophane P

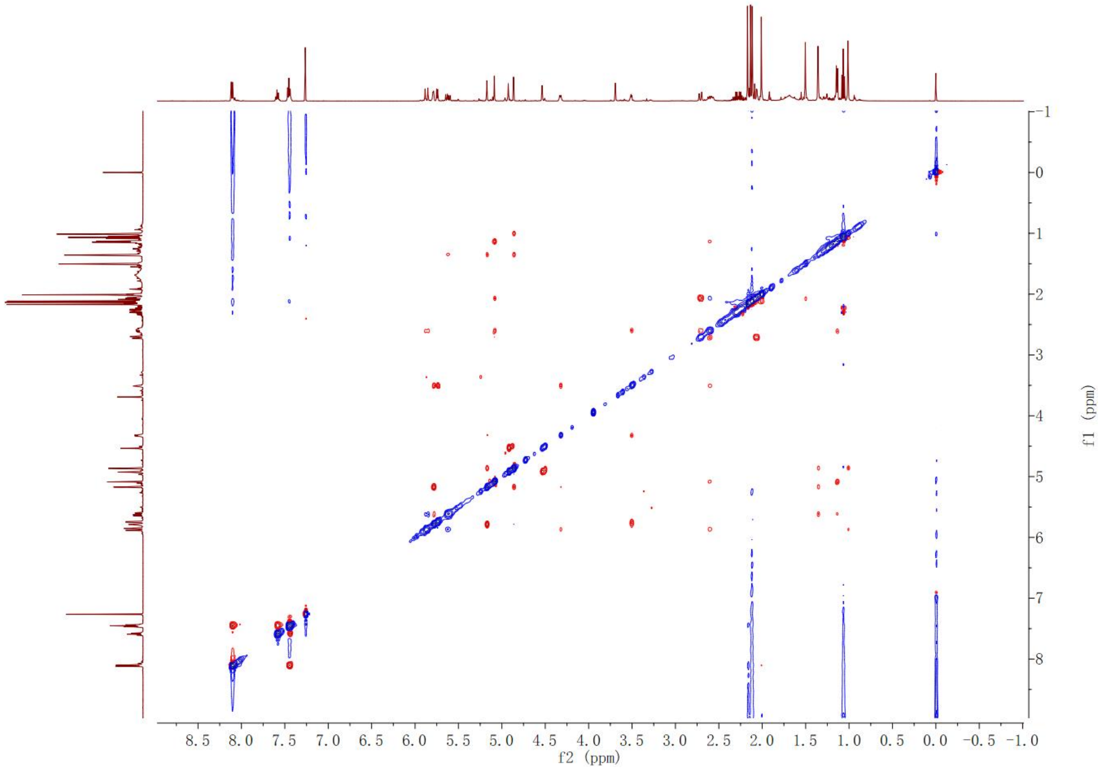

Figure S36. ROESY spectrum of Euphjatrophane P

Qualitative Analysis Report

**Data Filename**	h-gsp-30.d	**Sample Name**	h-gsp-30
**Sample Type**	Sample	**Position**	P1-A5
**Instrument Name**	Instrument 1	**User Name**	
**Acq Method**	s.m	**Acquired Time**	2/10/2023 11:20:39 AM
**IRM Calibration Status**	Success	**DA Method**	PCDL.m
**Comment**			

Sample Group **Info.**
Acquisition SW 6200 series TOF/6500 series
Version Q-TOF B.05.01 (B5125.2)

User Spectra

Fragmentor Voltage	Collision Energy	Ionization Mode
135	0	ESI

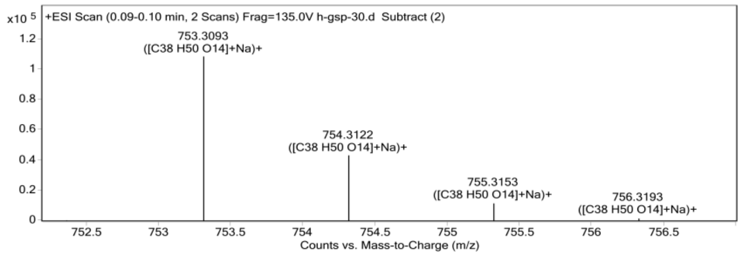

Peak List

m/z	z	Abund	Formula	Ion
713.3165	1	10145.14		
748.3543	1	199788.31		
749.3578	1	79312.49		
750.3594	1	20758.38		
753.3093	1	108623.38	C38 H50 O14	(M+Na)+
754.3122	1	43230.12	C38 H50 O14	(M+Na)+
755.3153	1	12000.38	C38 H50 O14	(M+Na)+
776.3545	1	25251.22		
777.3566	1	11398.99		
781.3035	1	18224.54		

Formula Calculator Element Limits

Element	Min	Max
C	3	60
H	0	150
O	0	20
N	0	3

Formula Calculator Results

Formula	CalculatedMass	CalculatedMz	Mz	Diff. (mDa)	Diff. (ppm)	DBE
C38 H50 O14	730.3201	753.3093	753.3093	0.00	0.00	14.0000

--- End Of Report ---

Figure S37. HR-ESI-MS spectrum of Euphjatrophane P

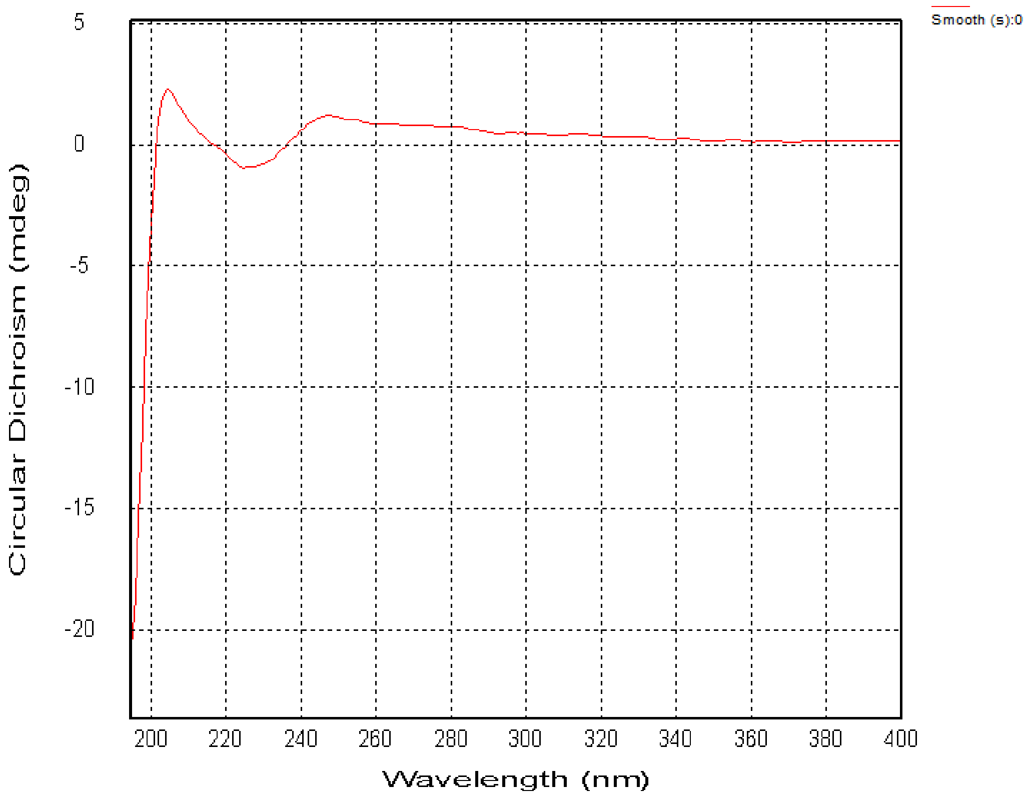

Figure S38. CD spectrum of Euphjatrophane P

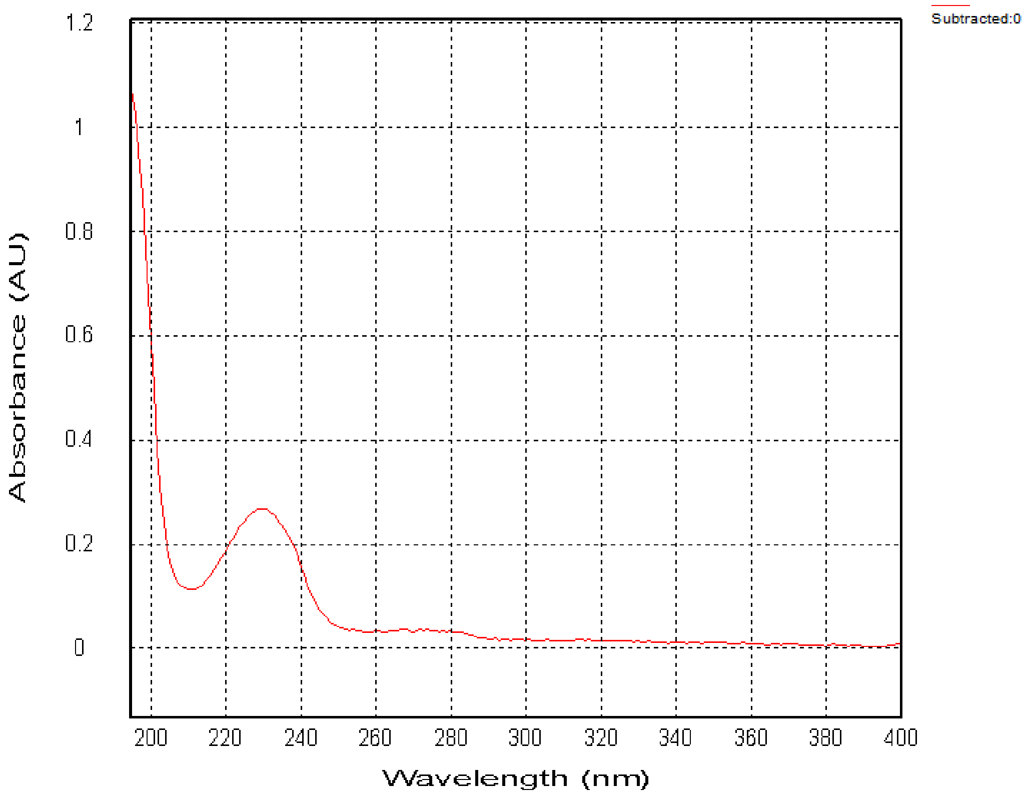

Figure S39. UV spectrum of Euphjatrophane P

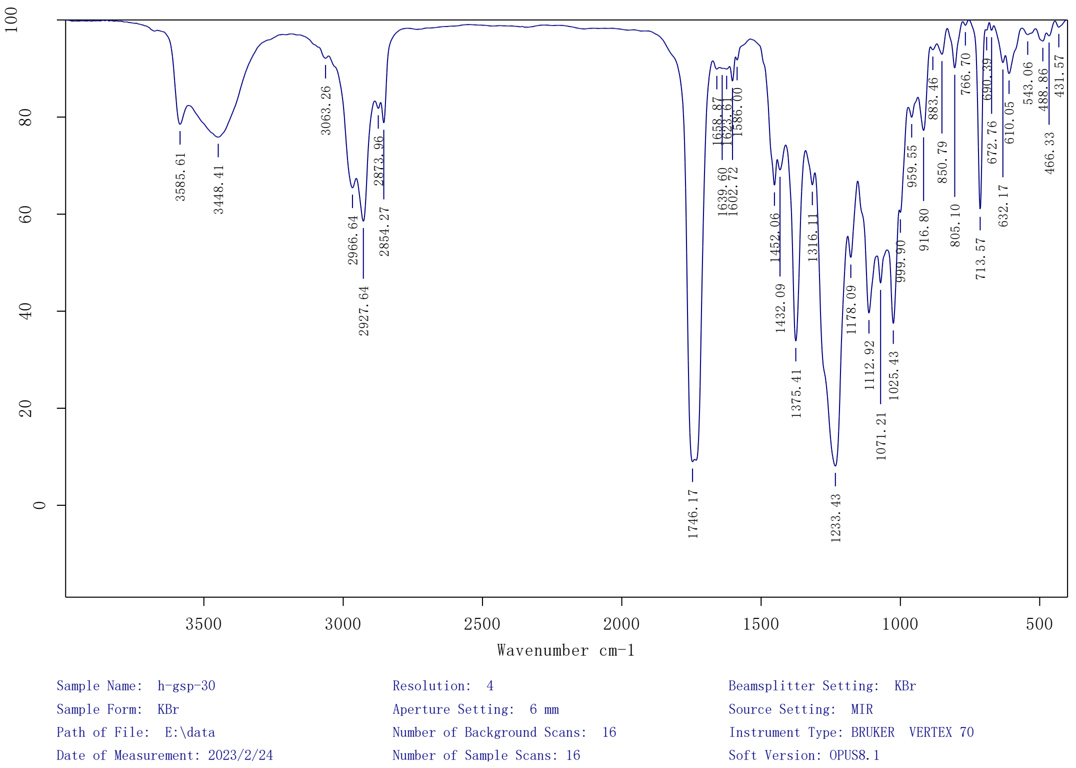

Figure S40. IR spectrum of Euphjatrophane P

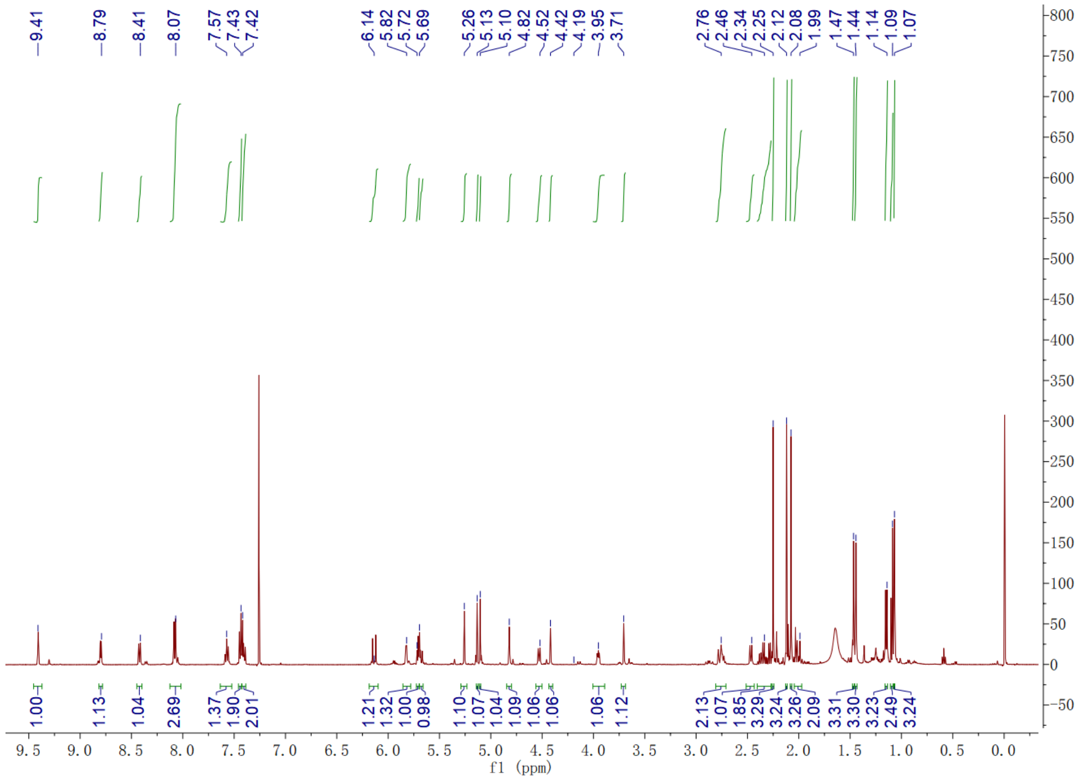

Figure S41.^{1}H-NMR spectrum of Euphjatrophane Q

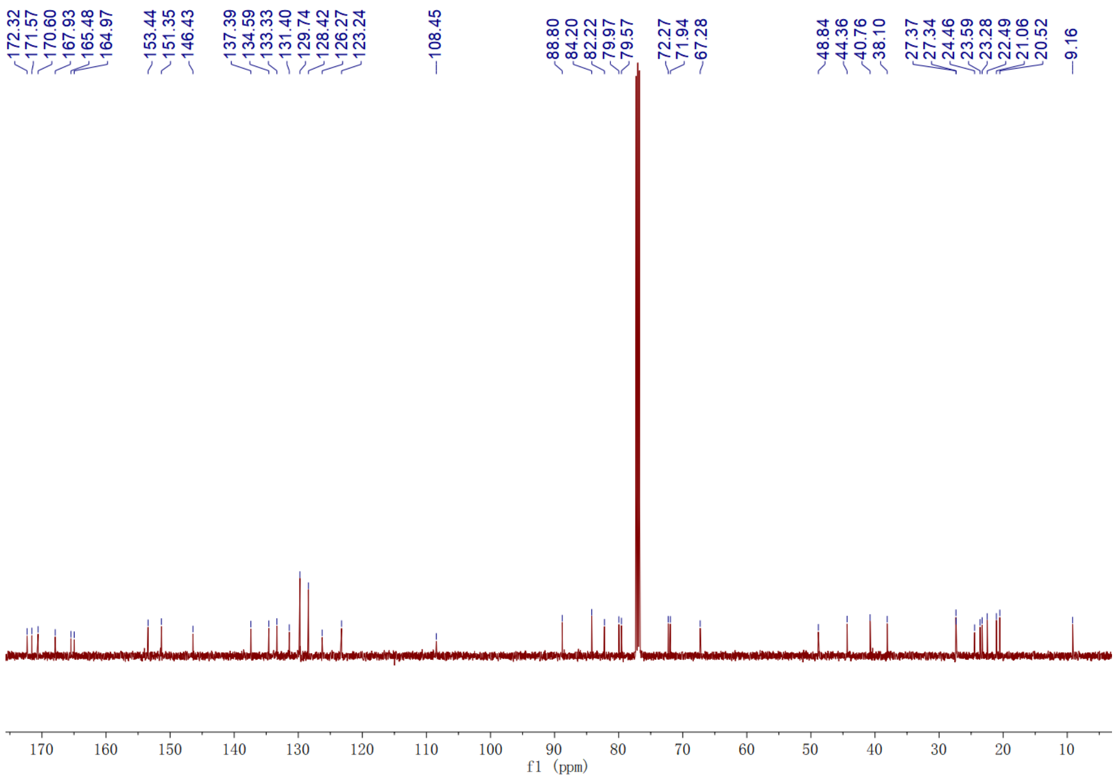

Figure S42.^{13}C-NMR spectrum of Euphjatrophane Q

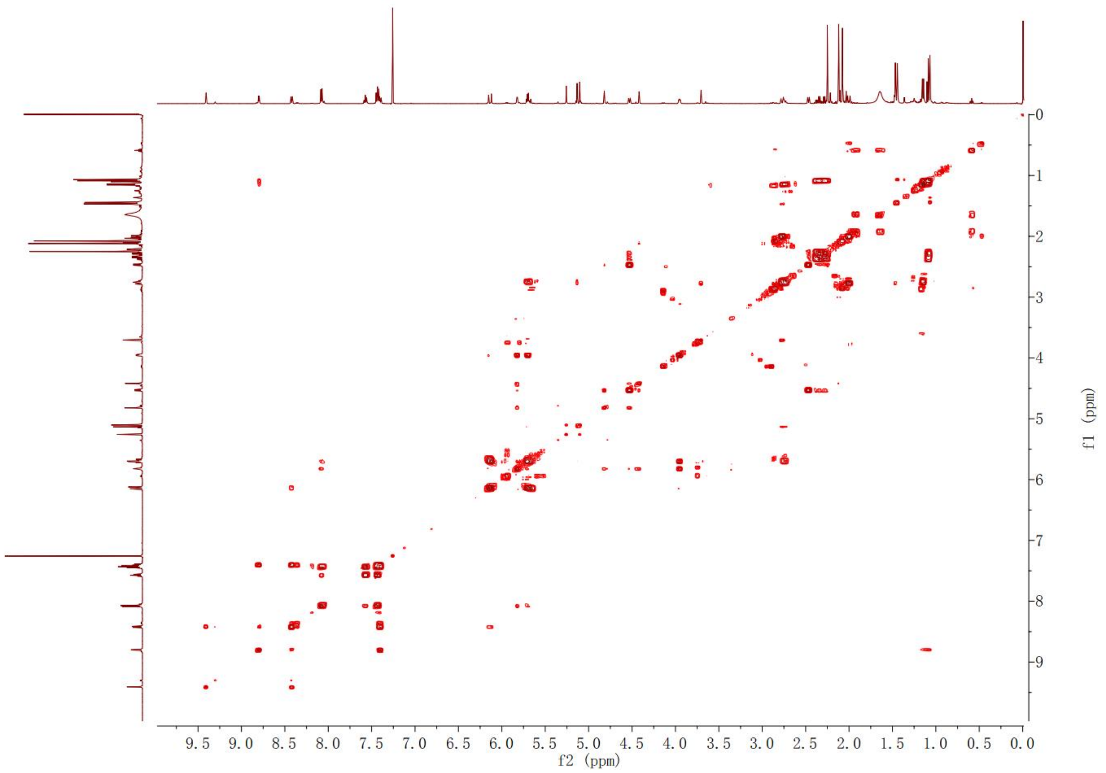

Figure S43.^{1}H-^{1}H COSY spectrum of Euphjatrophane Q

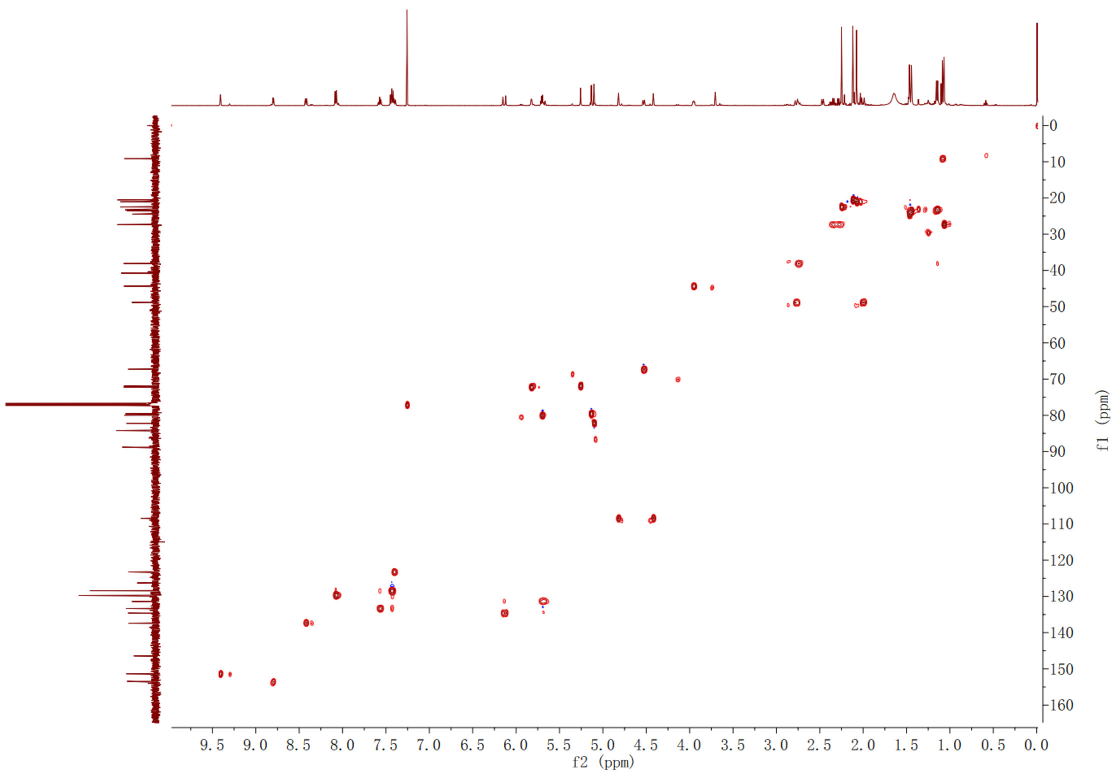

Figure S44. HSQC spectrum of Euphjatrophane Q

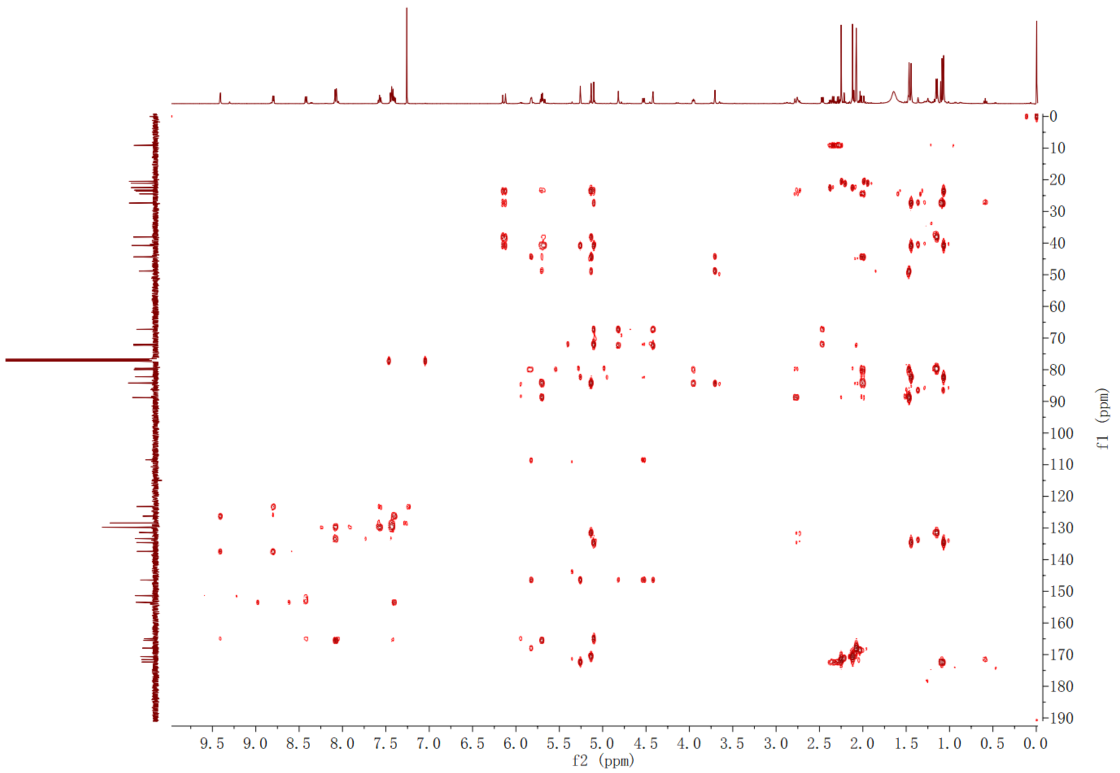

Figure S45. HMBC spectrum of Euphjatrophane Q

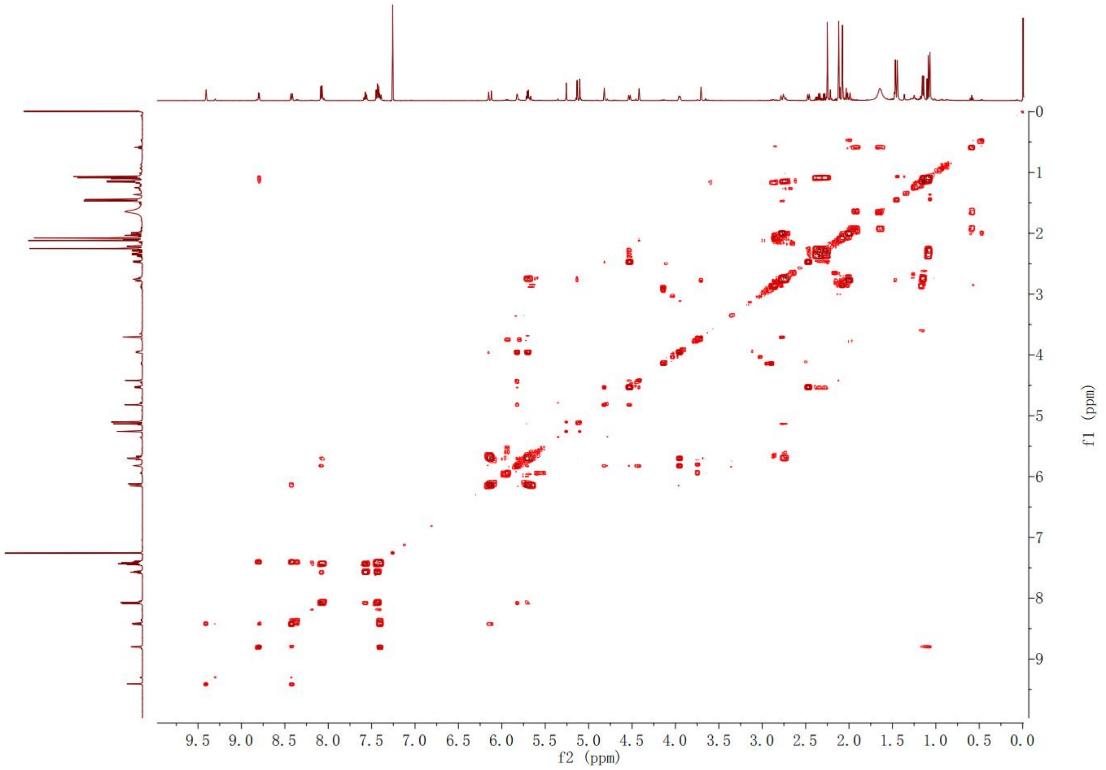

Figure S46. ROESY spectrum of Euphjatrophane Q

Qualitative Analysis Report

**Data Filename**	h-gsp-55.d	**Sample Name**	h-gsp-55
**Sample Type**	Sample	**Position**	P1-A8
**Instrument Name**	Instrument 1	**User Name**	
**Acq Method**	s.m	**Acquired Time**	2/10/2023 11:24:03 AM
**IRM Calibration Status**	Success	**DA Method**	PCDL.m
**Comment**			

Sample Group **Info.**

Acquisition SW Version 6200 series TOF/6500 series Q-TOF B.05.01 (B5125.2)

User Spectra

Fragmentor Voltage	Collision Energy	Ionization Mode
135	0	ESI

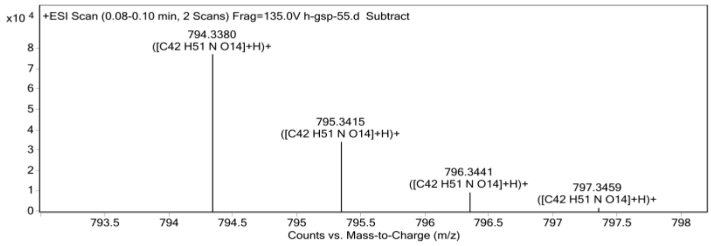

Peak List

m/z	z	Abund	Formula	Ion
706.3428	1	7348.51		
707.3469	1	2817.44		
711.2994	1	4570.35		
794.338	1	77420.73	C42 H51 N O14	(M+H)+
795.3415	1	34541.76	C42 H51 N O14	(M+H)+
796.3441	1	9852.52	C42 H51 N O14	(M+H)+
816.3199	1	36965.95		
817.3226	1	17456.34		
818.3256	1	5182.75		
839.3943	1	2618.45		

Formula Calculator Element Limits

Element	Min	Max
C	3	60
H	0	150
O	0	20
N	0	3

Formula Calculator Results

Formula	CalculatedMass	CalculatedMz	Mz	Diff. (mDa)	Diff. (ppm)	DBE
C42 H51 N O14	793.3310	794.3382	794.3380	0.20	0.25	18.0000

--- End Of Report ---

Figure S47. HR-ESI-MS spectrum of Euphjatrophane Q

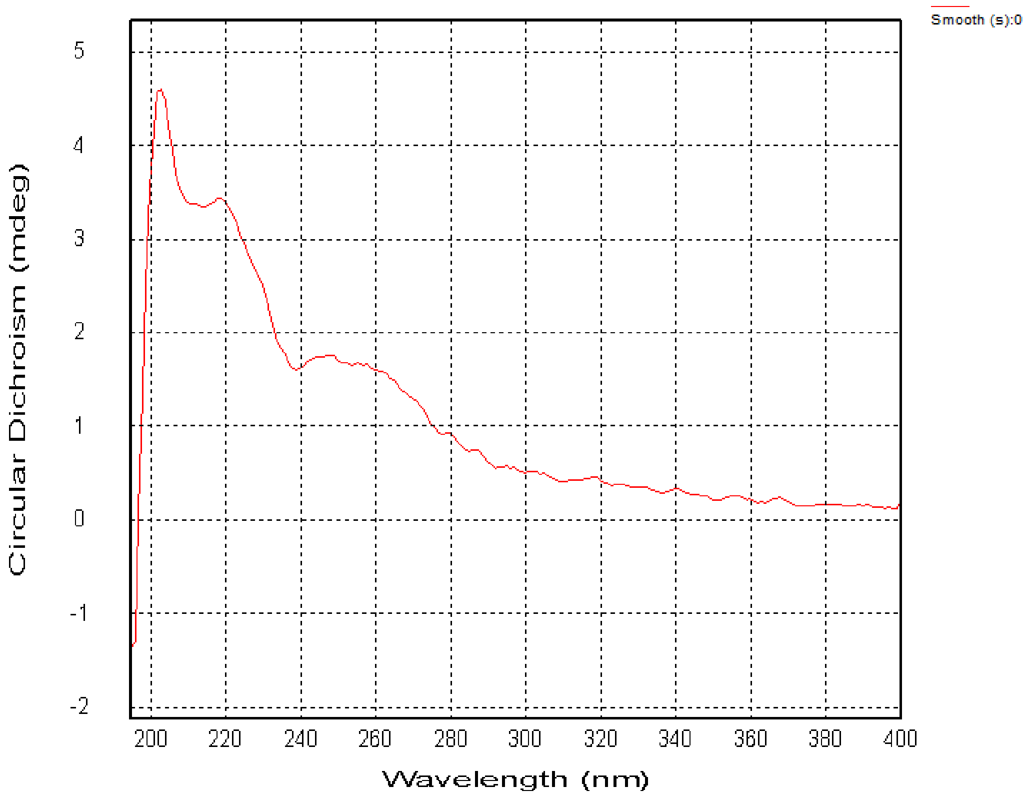

Figure S48. CD spectrum of Euphjatrophane Q

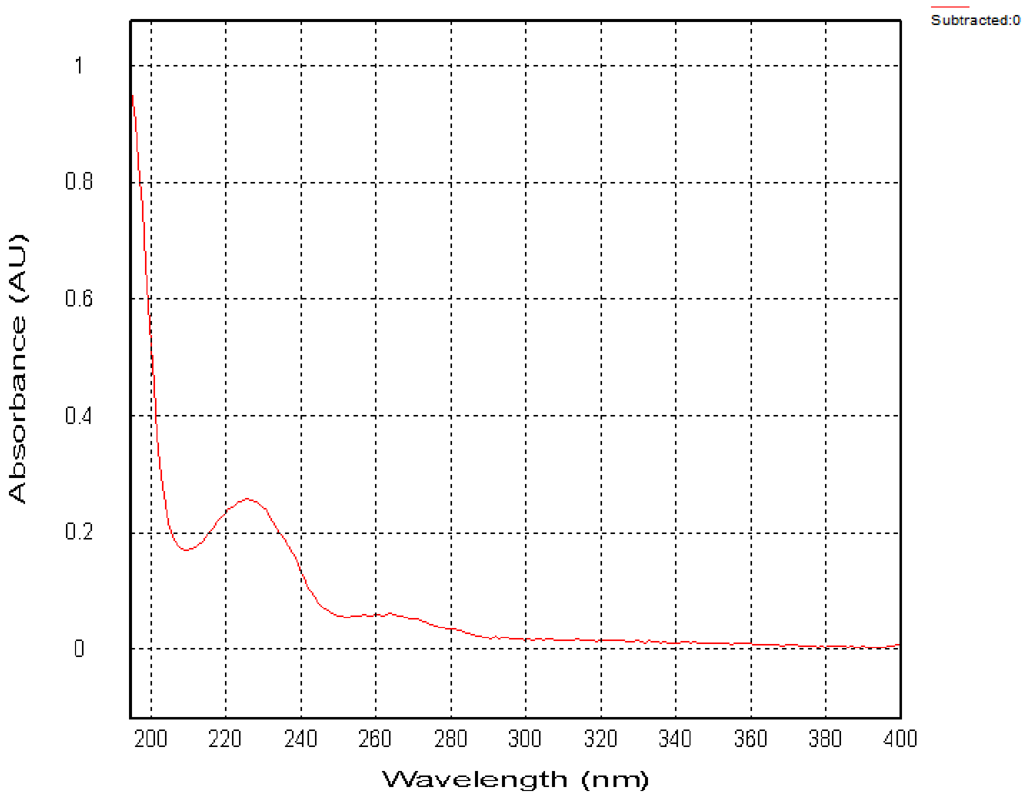

Figure S49. UV spectrum of Euphjatrophane Q

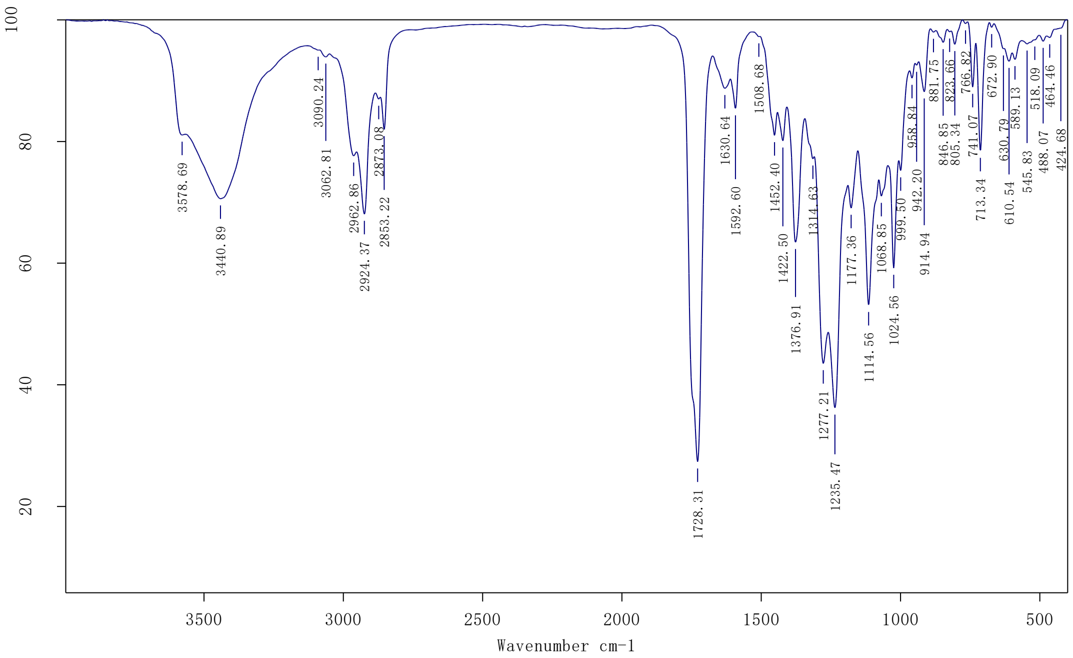

Sample Name: h-gsp-55
Sample Form: KBr
Path of File: E:\data
Date of Measurement: 2023/2/24
Resolution: 4
Aperture Setting: 6 mm
Number of Background Scans: 16
Number of Sample Scans: 16
Beamsplitter Setting: KBr
Source Setting: MIR
Instrument Type: BRUKER VERTEX 70
Soft Version: OPUS8.1

Figure S50. IR spectrum of Euphjatrophane Q
